# Interpretation of RNA Universe and Coding Potential Using *IntRNA*

**DOI:** 10.1002/advs.202509518

**Published:** 2025-08-22

**Authors:** Yunxia Wang, Minjie Mou, Shijie Huang, Wei Zhang, Ziqi Pan, Jing Tang, Yihao Wang, Qingxia Yang, Feng Zhu

**Affiliations:** ^1^ College of Pharmaceutical Sciences The Second Affiliated Hospital Zhejiang University School of Medicine State Key Laboratory of Advanced Drug Delivery and Release Systems Zhejiang University Hangzhou 310058 China; ^2^ Innovation Institute for Artificial Intelligence in Medicine of Zhejiang University Alibaba‐Zhejiang University Joint Research Center of Future Digital Healthcare Hangzhou 330110 China; ^3^ College of Basic Medicine Chongqing Medical University Chongqing 400016 China; ^4^ The High School Affiliated to Renmin University of China No. 37 Zhongguancun Street. Haidian District Beijing 100080 China; ^5^ Zhejiang Provincial Key Laboratory of Precision Diagnosis and Therapy for Major Gynecological Diseases Women's Hospital Zhejiang University School of Medicine Hangzhou 310058 China

**Keywords:** coding potential, deep learning, RNA annotation, RNA representation, RNA universe

## Abstract

The interpretation of *RNA universe* and coding potential are long‐standing issues in modern RNA studies, and three crucial questions remain unanswered: a) how to detect and interpret the coding potential of RNA, b) how to annotate the sophisticated taxonomy of the sncRNAs, and c) how to successfully distinguish between circular and linear lncRNAs. In this study, a multi‐channel deep learning framework, *IntRNA*, is thus constructed to interpret *RNA universe* and coding potential. First, a large number of RNA encoding features are proposed, which dramatically enlarged the available feature space. Second, a method realizing image‐like representation of RNA sequences is developed to describe the intrinsic correlation among the encoding features generated above. Third, a dual‐path model is constructed, which consistently performed the best among existing methods in various benchmarks. *IntRNA*’s interpretability is also validated by analysis, and all source codes are accessible at: https://idrblab.org/intrna/ and https://github.com/idrblab/intrna.

## Graphical abstract

This study constructs a multi‐channel deep learning framework *IntRNA* to interpret *RNA universe* and coding potential. *IntRNA* consistently performed the best among existing methods in various benchmarks. Moreover, *IntRNA*’s interpretability is also validated by real‐world analysis, which found some features indicating long‐distance contact between nucleobase pair to be critical in determining coding potential.

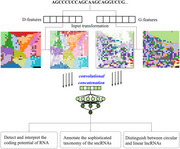

## 1 Introduction

**Figure 1:**
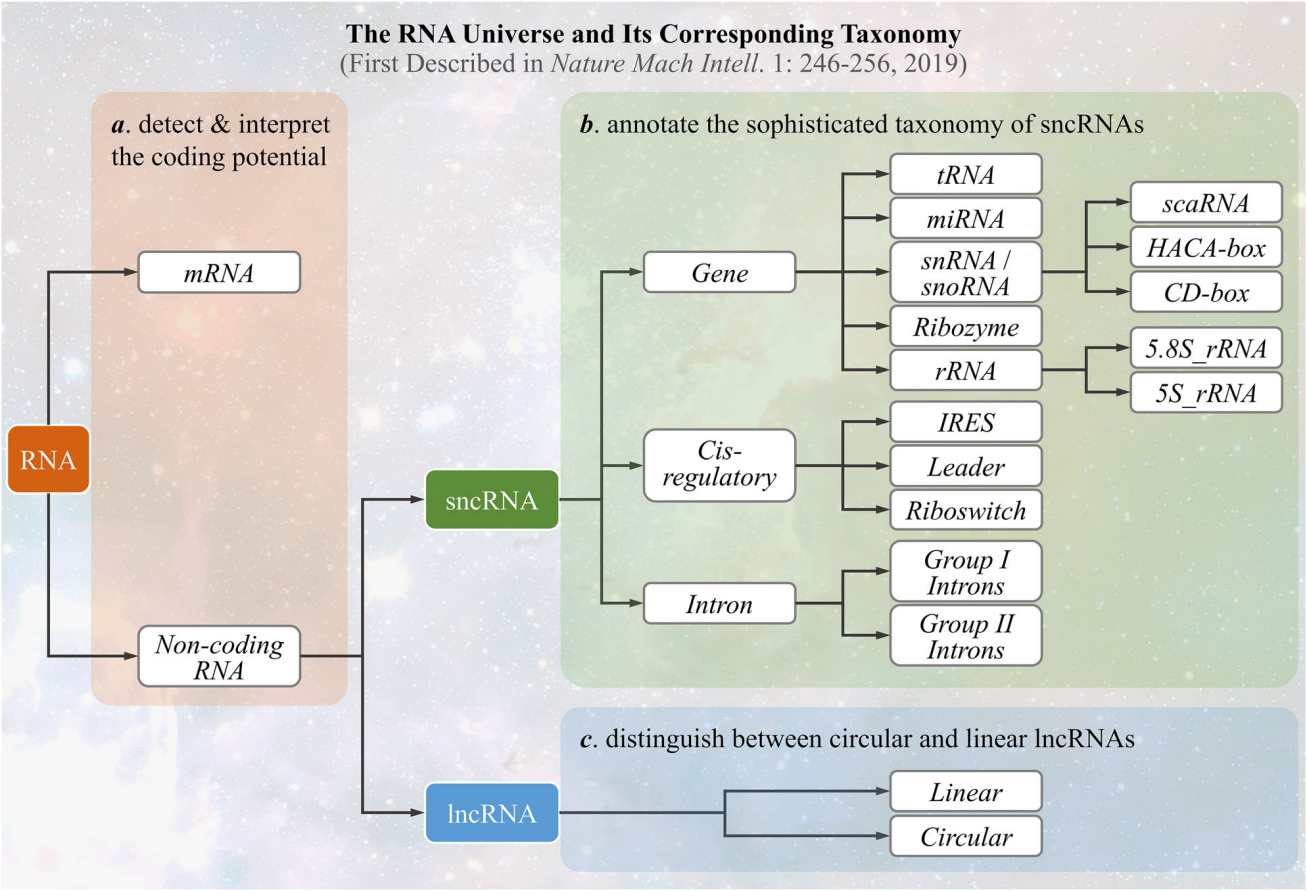
The *RNA universe* together with its corresponding taxonomy, which were first proposed by previous study (*Nature Mach Intell*. 1: 246‐256, 2019). To have a comprehensive understanding of the entire “*universe*”, three key problems remained to be answered: a) how to detect and interpret the coding potential of RNAs; b) how to annotate the sophisticated taxonomy of small non‐coding RNAs (sncRNAs); and c) how to successfully distinguish between circular and linear lncRNAs. Such problems (colored by light red, light green & light blue backgrounds) provided the key scientific questions that were planned to be answered in this research.

The annotation/classification of RNAs has emerged as a pivotal scientific challenge in advancing our understanding of sophisticated biological process and discovery of new therapeutic strategy.^[^ ^1^ ^]^ A comprehensive classification system of RNA has been proposed,^[^ ^2^ ^]^ which is frequently adopted by a variety of recent studies.^[^ ^3^, ^4^, ^5^ ^]^ In this system, an *RNA universe* is explicitly described (as shown in **Figure** **1**), which divides RNAs into messenger (mRNA) and non‐coding (ncRNA) ones based on their coding potentials.^[^ ^6^ ^]^ The ncRNAs are further categorized into small non‐coding (sncRNA, which provides sophisticated classification taxonomy) and long non‐coding (lncRNA, which can be further divided into linear and circular ones) ones based on their sequence length.^[^ ^7^ ^]^ For instance, the lncRNA HOTAIR identified through precise annotation drives cancer metastasis by modulating chromatin states,^[^ ^8^ ^]^ and deletion of the lncRNA Maenli locus on chromosome 2 causes severe congenital limb malformations.^[^ ^9^ ^]^

However, it remains extremely challenging to provide comprehensive and clear interpretation of the *RNA universe*.^[^ ^10^ ^]^ Particularly, three key problems (shown in Figure 1) still need to be answered: a) how to detect and interpret the coding potential of RNAs,^[^ ^11^ ^]^ b) how to annotate the sophisticated taxonomy of those sncRNAs,^[^ ^12^ ^]^ and c) how to successfully distinguish between circular and linear lncRNAs.^[^ ^13^ ^]^ Till now, a number of methods (derived from the classical machine learning strategy) have been constructed to achieve fast and accurate prediction of RNA coding potential based on sequence‐intrinsic (such as CPPred,^[^ ^14^ ^]^ CPAT,^[^ ^15^ ^]^ COME,^[^ ^16^ ^]^ and mRNN^[^ ^17^ ^]^), nucleotide counts‐based (such as PLEK,^[^ ^18^ ^]^ RNAmining,^[^ ^19^ ^]^ and DeepCPP^[^ ^20^ ^]^) and physicochemical properties‐directed (such as CPC2,^[^ ^21^ ^]^ CPE‐SLDI,^[^ ^22^ ^]^ and NCResNet^[^ ^23^ ^]^) encoding descriptors of the ribonucleic acids.

Moreover, some tools are available for depicting the sncRNA taxonomy (such as nRC,^[^ ^24^ ^]^ ncRNA‐deep,^[^ ^25^ ^]^ GraPPLE,^[^ ^26^ ^]^ and EDeN^[^ ^27^ ^]^) based on various characteristics (including secondary structure, atoms‐based graph, etc.), and some others are constructed to discriminate circular lncRNAs from linear ones (such as circDeep,^[^ ^28^ ^]^ CirRNAPL,^[^ ^29^ ^]^ PredcircRNA,^[^ ^30^ ^]^ and StackCirRNAPred^[^ ^31^ ^]^) through fusing various types of sequence‐intrinsic descriptor. Meanwhile, researchers have leveraged the language models to elevate the ability in RNA classification (such as RNA‐MSM,^[^ ^32^ ^]^ RNAErnie,^[^ ^33^ ^]^ and RNABERT^[^ ^34^ ^]^), which offer additional choices on the interpretation of *RNA universe*.

As reported, the relation among the nucleotides of “long‐distance interval” can largely determine RNAs’ function annotation by shaping their structure,^[^ ^35^ ^]^ but none of the methods discussed above has taken such critical features into consideration when encoding the *RNA universe*. Additionally, the performances of the latest Artificial Intelligence (AI) techniques are found susceptible to the order of studied features in their representation,^[^ ^36^ ^]^ which asks for the construction of new strategy for capturing the innate relationship among RNA encoding features. Finally, there is a significant lack of interpretability in the available RNA annotation tools,^[^ ^37^, ^38^ ^]^ which hinders the provision of new biological insight for modern RNA research. In other words, there remains substantial rooms for improvement in the classification of *RNA universe* and the interpretation of RNA annotations (especially, in the explanation of the mechanism underlining RNA's coding potential).

In this study, a multi‐channel deep learning framework, entitled *IntRNA*, was therefore developed to enable the annotation of *RNA universe* and interpretation of coding potentials with extensively elevated model performance. First, a large number of new encoding features (especially the ones describing the nucleotides of long‐distance interval) were proposed, which dramatically enlarged (to over four times) the existing feature space that encodes RNAs. Second, a method that realized image‐like representation of RNA sequences was constructed to describe the intrinsic correlation among the massive amount of those encoding features generated above. Third, to further enhance the interpretability of both *RNA universe* and coding potentials, a dual‐path multi‐channel model was developed, which was found consistently well‐performing when comparing with the existing methods in a series of benchmark studies. Moreover, the interpretability of *IntRNA* was validated by a real‐world study, which identified key structural features determining RNA coding potential. All in all, our *IntRNA* was freely accessible at: https://idrblab.org/intrna/, and all the source codes were readily downloadable from GitHub at: https://github.com/idrblab/intrna.

## 2 Results and Discussion

### 2.1 The Framework Proposed in This Research and Its Characteristics

**Figure 2:**
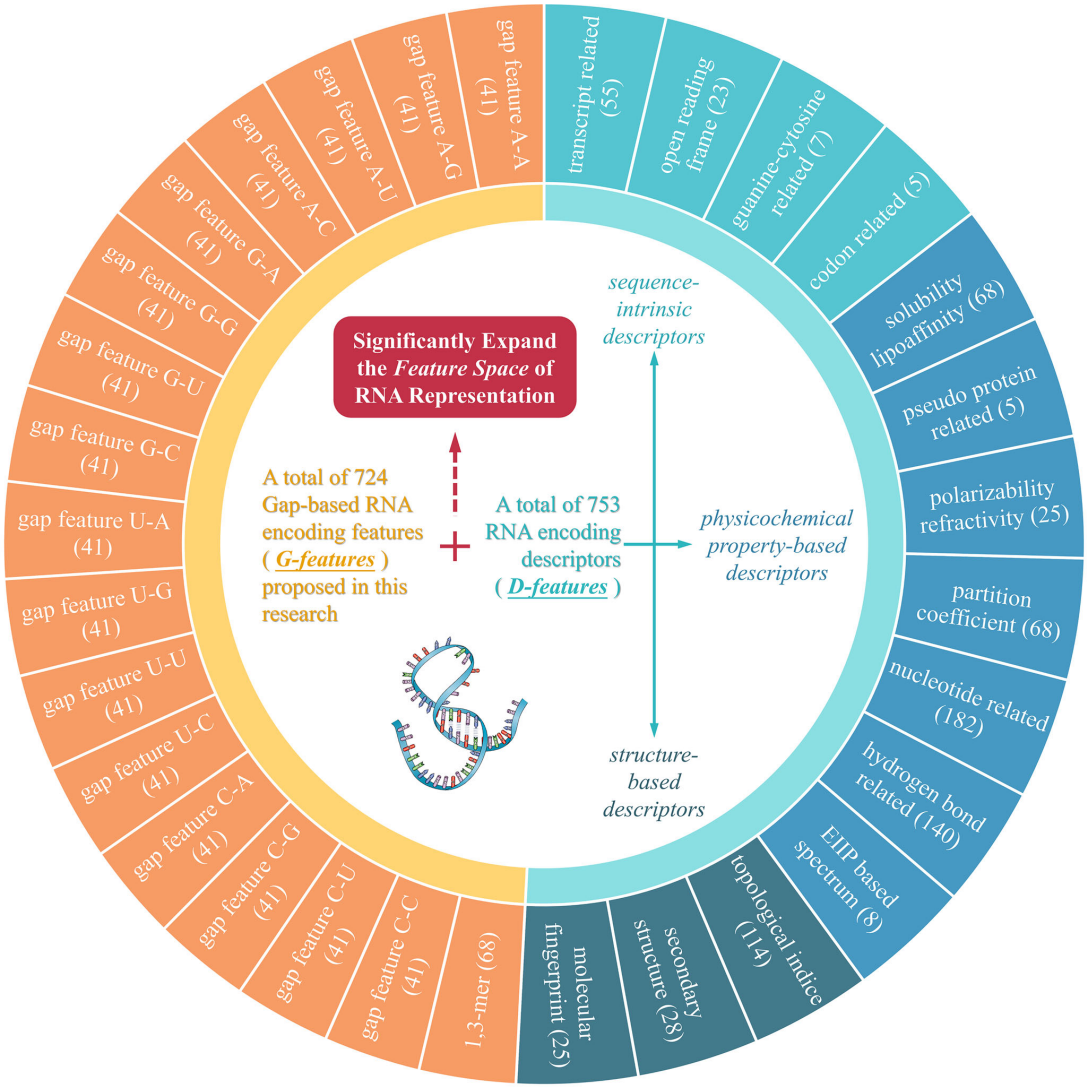
The significantly expanded feature space for RNA representation. There were two types of encoding features: the gap‐based RNA encoding features (*G‐features*, a total of 724 features fully proposed in this study) and the RNA encoding descriptors (*D‐features*, a total of 753 features, 439 (58%) out of which were the newly‐proposed ones). For both types, their corresponding feature subgroups (17 groups and 14 groups for *G‐features* and *D‐features*, respectively) were provided in the outer‐most leaves, and the 14 subgroups of *D‐features* were further summarized to three classes (sequence‐intrinsic, structure‐based and physicochemical property‐based). The number in each bracket indicated the total number of features in each feature subgroup.

**Figure 3:**
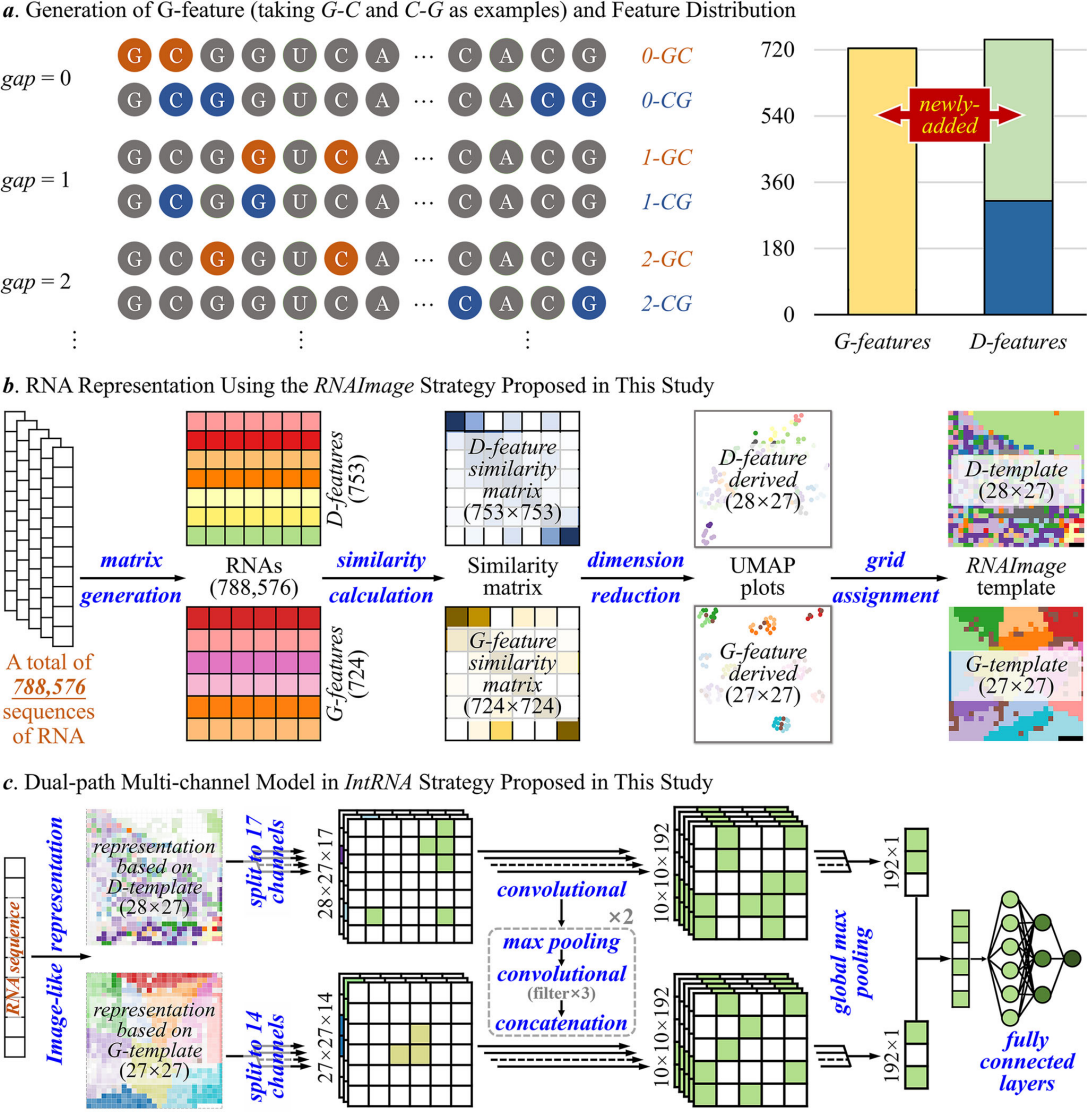
The framework of the *IntRNA* strategy proposed in this research. a) the generation of *G‐feature* and feature distribution. New features for encoding the long‐distance relations between nucleobases were proposed. Taking *G‐C* and *C‐G* as examples, the *G‐features* were generated based on the gapped distances (from 0 to 40, described on the left side). A total of 724 *G‐features* and 439 *D‐features* proposed in this research, which were further enriched by 314 previously‐reported *D‐features*, were adopted to significantly expand the feature space of RNA representation (provided on the right side). b) RNA representation using *RNAImage* method proposed in this study. Two template images (*D‐template* and *G‐template*) were generated using a total of 788576 RNA sequences collected from two established databases (*RefSeq* and *ENCODE*) based on four sequential processes (from matrix generation, to similarity calculation, then to dimension reduction, and finally to grid assignment). c) *IntRNA* strategy proposed in this study for interpreting *RNA universe*. Each RNA sequence was represented as two images of different matrix dimensions (28 × 27 & 27 × 27) based on the newly‐generated template images, and the image‐like representations of all RNAs in a studied key problem were adopted to construct a dual‐path multi‐channel model, which was explicitly described above and in the section of Methods.

The *IntRNA* was constructed here to enable the annotation of *RNA universe* and interpretation of coding potential with enhanced performance. To achieve such goal, the most comprehensive list of encoding features (especially the ones describing the nucleotides of long‐distance interval, as provided in **Figure** **2**) among the existing methods were first produced to extensively expand (to over four times, as shown on the right side of **Figure** **3**a) the feature space of RNA representation. Second, a novel method that realized the image‐like representation of RNA sequences (illustrated in Figure 3b) was developed to provide the intrinsic correlations among encoding features. Third, a new model was further constructed based on a dual‐path multi‐channel deep learning algorithm (shown in Figure 3c). Furthermore, the results of ablation study on *IntRNA* model were given in Figure S1 (Supporting Information). Compared with the performances of the latest *IntRNA* model (which were measured by Matthews correlation coefficient), that of the ablated models (the one deprived of *G‐features*, the one deprived of *D‐features*, and the one deprived of *RNAImage*) decreased by 25.6%, 4.9%, 18.3%, respectively. All in all, such results highlighted the critical contribution of both the *G‐features* and the *RNAImage* strategy proposed in this study.

### 2.2 Performance Comparison in Solving the Problems of *RNA Universe*

**Figure 4:**
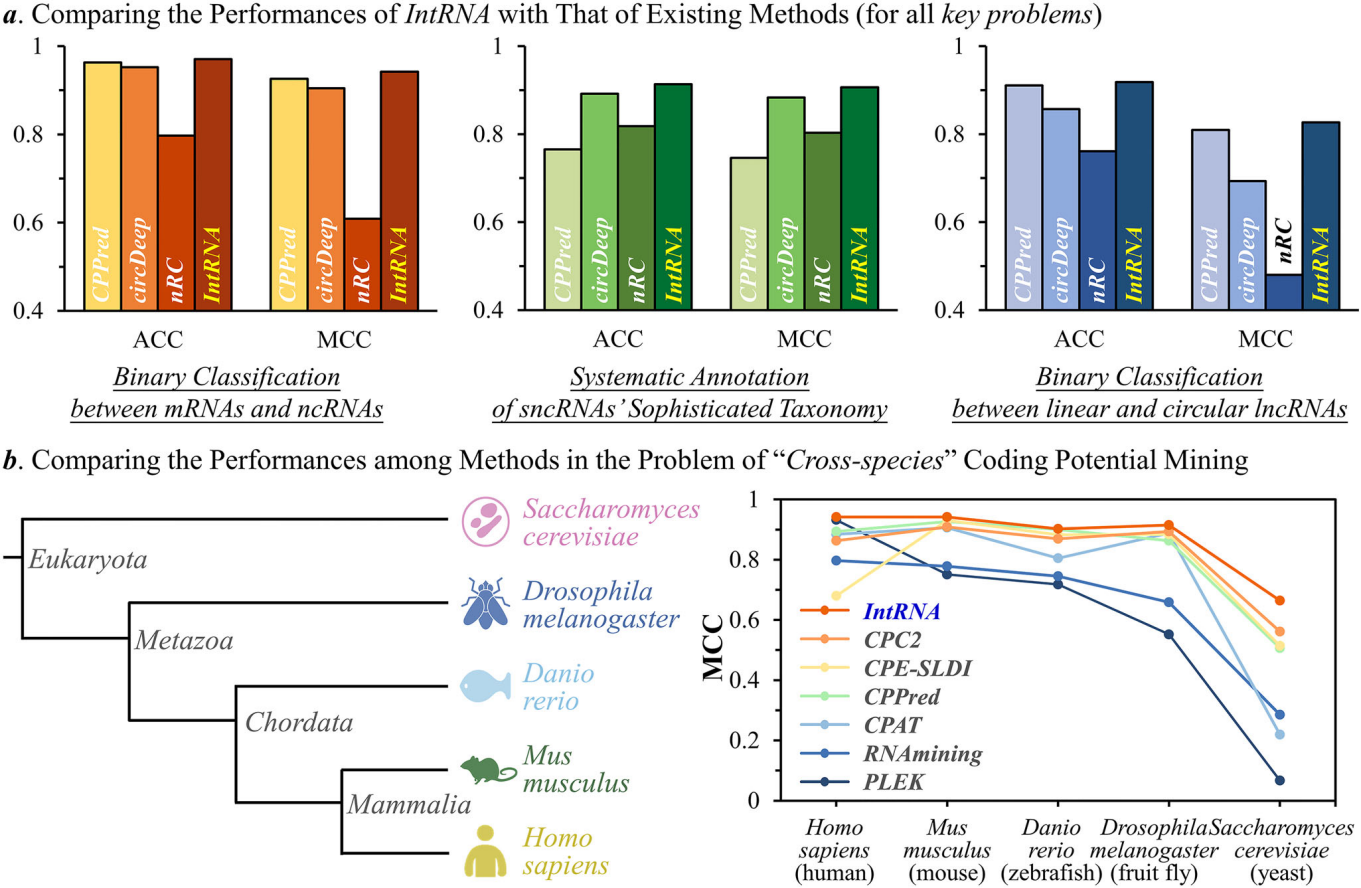
Performance comparison with existing methods and in the problem of cross‐species coding potential minding. a) performance comparison among *IntRNA* and three existing methods (*CPPred*, *circDeep* and *nRC*). The performances of all four methods when solving three key problems (as provided in Figure 1) were assessed, and *IntRNA* was identified consistently best‐performing among four methods. b) comparing the performances of *IntRNA* and six existing methods that were designed to mine the coding potential of RNAs. The evolutionary divergence between human (*homo sapiens*) and other four typical species (described on the left side) increased progressively. The performances of all seven methods were systematically assessed using the RNAs from four typical species (by training their models using the human RNAs, described on the right side), and *IntRNA* was identified consistently best‐performing in all species. Particularly, with the increase of evolutionary divergence, the *IntRNA* demonstrated much better performance enhancement comparing with other six methods.

As provided in Figure 1, the classification system required the solving of three key problems: a) how to detect and interpret RNA coding potential, b) how to annotate the sophisticated taxonomy of sncRNAs, and c) how to successfully distinguish between circular and linear lncRNAs. Based on our literature review, *CPPred* was constructed for classifying mRNA and ncRNA,^[^ ^14^ ^]^ *nRC* was made available to classify sncRNAs,^[^ ^24^ ^]^ and *circDeep* was constructed for differentiating between circular and linear lncRNAs.^[^ ^28^ ^]^ Therefore, the performances of those three popular methods were compared with that of the newly‐constructed *IntRNA*. As provided in Table S1 (Supporting Information), the performances of the three existing tools (*CPPred*, *circDeep* and *nRC*) alongside *IntRNA* were compared based on five metrics, and the results of this comparison were described in **Figure** **4**a. As illustrated, the bar plots of accuracy (ACC) and Matthews correlation coefficient (MCC) were utilized to give the performances of four methods. As a result, *IntRNA* consistently outperformed three existing tools in all key problems. Specifically, as observed in Figure 4a, *IntRNA* performed as well as *IntRNA* in the “binary classification between mRNAs and ncRNAs” (this is the problem what *CPPred* was constructed for). Similarly, *IntRNA* performed as well as the remaining two existing models on their intended task. While CPPred, circDeep, and nRC were originally developed for addressing specific RNA classification tasks, the comparative analysis is intended to demonstrate general applicability of the *IntRNA*.

### 2.3 Performance Comparison in Mining Cross‐Species Coding Potential

The mining of RNA coding potential has been considered as a long‐standing challenge in modern RNA research,^[^ ^39^, ^40^, ^41^ ^]^ and several methods have therefore been constructed (the representative tools under this category were offered in Introduction section). However, the mining of cross‐species coding potentials remained a huge challenge,^[^ ^42^ ^]^ which asked for the construction of new powerful tool to address this critical issue. In this study, the performances of *IntRNA* and six existing tools (currently reproducible with source code provided) were thus compared based on the benchmark dataset provided in Table S2 (Supporting Information). As demonstrated on the left side of Figure 4b and Table S3 (Supporting Information), the evolutionary divergence between human (*homo sapiens*) and four typical species increased progressively, and the data used in this research to assess seven methods’ performances in the detection and interpretation of RNAs’ coding potential had a total of 180233 RNAs (including 112726 mRNAs & 67507 ncRNAs) from five different species: *Homo sapiens*, *Mus musculus*, *Danio rerio*, *Drosophila melanogaster* & *Saccharomyces cerevisiae*. Particularly, the data from *Homo sapiens* were split by the original publication^[^ ^14^ ^]^ into the training (comprising 33360 mRNAs and 24163 ncRNAs) and the test (comprising 8557 mRNAs and 8241 ncRNAs) datasets. During the training process, the strategy of fivefold cross‐validation was applied, and the test dataset was further enriched by integrating the data from the remaining four species.

As shown on the right side of Figure 4b, the performances of seven methods (shown in Table S4, Supporting Information) were systematically evaluated using the RNAs from four typical species, and *IntRNA* was identified consistently best‐performing for all species. Particularly, with the increase of evolutionary divergence, the *IntRNA* provided much better performance enhancements comparing with other six methods. Particularly, a detailed comparison among the performances of seven methods was offered in Table S5 (Supporting Information). As described using two important metrics (AUC & MCC), the performance of *IntRNA* consistently outperformed the existing tools (highlighted using BOLD fonts), which underscored the good performance of *IntRNA* in mining the cross‐species RNA coding potential. Besides the animal datasets, *IntRNA* also achieved good performance on a plant dataset, such as Arabidopsis thaliana (ACC = 0.9101, MCC = 0.8324, AUC = 0.9782), further demonstrating its broad cross‐species applicability. This comparison highlights *IntRNA*’s generalization ability and is not intended to critique tools optimized for species‐specific retraining.

### 2.4 Discovery of the RNA Coding Potential Based on Feature Ranking

**Figure 5:**
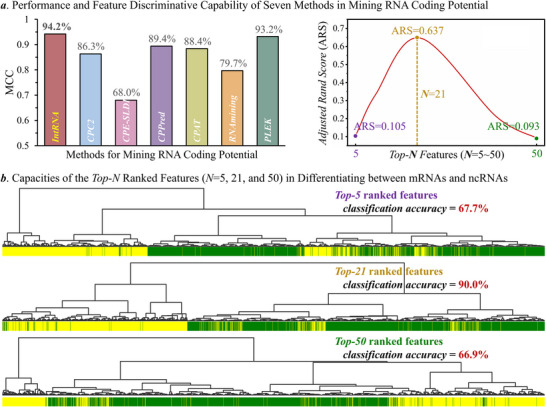
Performance comparison among seven methods designed to mine the coding potential of RNAs (by training their models using a benchmark dataset collected from *CPPred*, which contained 74321 human RNAs, and the way adopted here to split training and test datasets was exactly the same as that described in the original publication of *CPPred*). a) performance and feature discriminative capability of each method for mining RNA coding potential. Particularly, the values of matthews correlation coefficient (MCC) were used to measure the method performance (described on the left side), and the values of adjusted rand score (ARS) were utilized to measure the capacity of the *top‐N* ranked features (*N* = 5∼50) in detecting RNA coding potential (shown on the right side), which identified that the *top‐21* ranked features performed the best. b) the capacities of the *top‐N* ranked features (*N* = 5, 21, and 50) in differentiating between mRNAs and ncRNAs. The resulting classification accuracies for *top‐5*, *top‐21*, and *top‐50* features were 67.7%, 90.0%, and 66.9%, respectively, which indicated the good differential capacity of those *top‐21* ranked features that were identified in this analysis.

The performance of *IntRNA* in mining coding potential could also be reflected by the left side of **Figure** **5**a. As shown, the MCC value of *IntRNA* was significantly higher than that of other tools, which reached 94.2% and was higher than that (ranging from 68.0% to 93.2%) of available tools. To test the ability of *IntRNA* in capturing the critical features that endorsed RNA coding potential, a prediction model was first trained using the human coding potential dataset, and the importance of each feature was measured using permutation algorithm (as described in the Methods section), which resulted in the ranking of all features based on their importance. Second, the adjusted rand scores (ARSs) were calculated for measuring the ability of the top‐*N* ranked features (*N* = 1∼1477) to discover the coding potential.^[^ ^43^ ^]^ The higher the ARS value was, the better ability of the top‐*N* ranked features was in identifying RNA coding potential. As provided on the right side of Figure 5a, the top‐21 features (shown in Table S6, Supporting Information) were found to provide the highest ARS value, which highlighted the great contribution of these features. Third, the capacity of these features in discriminating mRNA from ncRNA was further demonstrated in Figure 5b. As described, the abilities of the top‐*N* (*N* = 5, 21 and 50) ranked features in discovering coding potential were evaluated by hierarchical clustering, and mRNAs and ncRNAs were colored in GREEN and YELLOW, respectively. As a result, the classification accuracies of top‐5, top‐21, and top‐50 features equaled to 67.7%, 90.0% and 66.9%, respectively, which further emphasized the importance of the identified top‐21 features.

The role of identified features in coding potential prediction was also investigated. As shown in Figure S2a (Supporting Information), there were clear correlations between the features from the training dataset and that from the test one. Particularly, the Pearson's correlation coefficients of *D‐feature* and *G‐feature* reached 0.993 and 0.968, respectively. Moreover, an in‐depth analysis on the best‐ranking features (based on their importance score *IS* > 0.8, shown in Methods) was provided in Figure S2b (Supporting Information) for *D‐feature* (on the left side) and *G‐feature* (on the right side). If the *IS* was larger than 0.8, the corresponding feature was then highlighted. For *D‐features*, “open reading frame”, “EIIP based spectrum”, “hydrogen bond” and some other groups were identified, and some of the identified groups had been reported to be closely related to RNA coding potential. For example, the open reading frame was frequently adopted to enable sequence‐based discovery of mRNAs;^[^ ^44^ ^]^ the EIIP based spectrum had been applied to differentiate mRNAs from ncRNAs;^[^ ^45^ ^]^ the hydrogen bond was found helpful in facilitating coding potential estimation.^[^ ^46^ ^]^ For *G‐features* (right side of Su Figure S2b, Supporting Information), the numbers in squares indicated the amount of bases between the studied base pair, and the numbers of long‐distance (number≥20) and short‐distance (number<20) *G‐features* were colored by YELLOW and BLACK, respectively. It was clear that the amount of long‐distance features (with yellow‐colored number) was significantly larger than that of the short‐distance ones, which indicated the great contribution of “long‐distance contacts” between base pairs. Similar conclusion had been identified by previous work, which emphasized the importance of certain long‐distance segments in the determination of RNA function.^[^ ^47^ ^]^

To demonstrate the generality of *IntRNA*’s interpretability, the feature importance analysis was extended to the other two key problems: how to annotate the sophisticated taxonomy of sncRNAs (shown in Figures S3 and S4, Supporting Information), and how to successfully distinguish between circular and linear lncRNAs (presented in Figure S5, Supporting Information) using the same permutation‐based ranking approach. In the sncRNAs annotation, ribozyme RNAs showed enrichment in topological indices and partition coefficients, reflecting their catalytic folding and solvent‐sensitive architecture.^[^ ^48^, ^49^ ^]^ Introns were marked by motif‐level correlation features and entropy‐based metrics, consistent with splicing signals and non‐coding character.^[^ ^50^, ^51^, ^52^ ^]^ To distinguish circular and linear lncRNA, top features included hydrogen bond donors/acceptors and solubility‐related descriptors, which are consistent with the compact, base‐paired, and stable nature of circular RNAs.^[^ ^53^, ^54^, ^55^ ^]^ This comprehensive analysis highlights *IntRNA*’s ability to extract meaningful, task‐specific features across RNA classes, reinforcing its utility not only as a high‐performance classifier but also as a tool for uncovering mechanistic insights in RNA biology.

### 2.5 In‐Depth Interpretation of Coding Potential by Molecular Example

**Figure 6:**
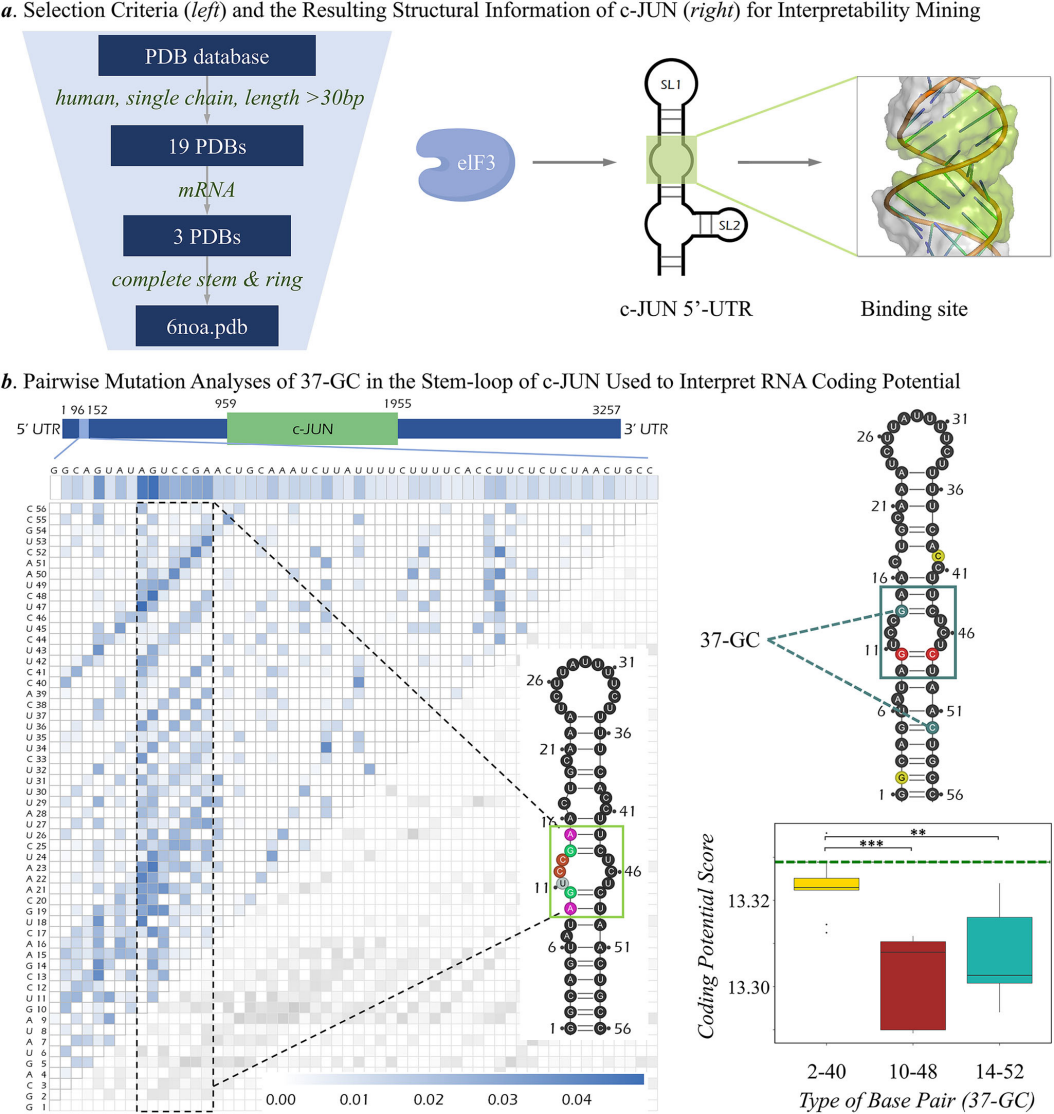
Interpretation of RNA coding potential by taking c‐JUN as an example. a) selection criteria and the resulting structural information of c‐JUN. The stem‐loop highlighted in GREEN of c‐JUN 5′‐UTR mRNA was the key structure recognized by eIF3. b) pairwise mutation analyses of 37‐GC in the stem‐loop of c‐JUN used to interpret RNA coding potential. The pairwise mutation analysis was conducted on c‐JUN's 5′‐UTR segment (containing 56 base pairs) to ensure the impact score of each base pair on RNA coding potential (described on the left side). 37‐GC was found at three different positions (2‐40, 10‐48 and 14‐52) in 5′‐UTR region (as shown in the upper right side), and a boxplot of coding potential scores was applied for visualizing the importance of 37‐GC found at different locations (2‐40, 10‐48 and 14‐52) (provided in the lower right side). The green dashed line showed the coding potential scores of the original c‐JUN mRNA sequence. The *p*‐value <0.01 was denoted using two asterisks (**), while the *p*‐value <0.001 were indicated using three asterisks (***).

To assess the interpretability of *IntRNA*, specific RNA structure was adopted to validate the RNA coding potential prediction. First, RNA structures were filtered from PDB database by following the criteria provided on the left side of **Figure** **6**a, which identified one RNA sequence (PDB ID: 6NOA, c‐JUN 5′‐UTR mRNA). A stem‐loop structure in this mRNA was identified (colored in GREEN on the right side of Figure 6a), which had been reported to be crucial for the recognition of this mRNA by protein eIF3 and for the specialized translation initiation of c‐JUN.^[^ ^56^ ^]^ eIF3 directly binds the stem‐loop structure within the c‐JUN 5′ untranslated region (5′ UTR), which is essential for specialized c‐JUN translation and the dysregulation disrupts the precise control of c‐JUN expression.^[^ ^57^ ^]^

On the left side of Figure 6b, a pairwise mutation analysis was performed on the c‐JUN 5′‐UTR segment (56 bp) using *IntRNA* to assess the contribution of any studied nucleobase pair to coding potential of RNA. The darker the color of a cell was, the greater contribution of the corresponding nucleobase pair to coding potential was. As described, the bases within the location from 9 to 15 offered the most significant contribution to the coding potential, which perfectly overlapped with the stem‐loop structure highlighted in GREEN on the right side of Figure 6a and the left side of Figure 6b. Furthermore, the contribution to coding potential was different for pairwise mutations of the same *G‐features* when a studied nucleobase pair was found in different sequence locations. Taking the gap‐feature *G‐C* as an example, its *G‐feature* of 37 bases gap (37‐GC) was discovered as important (*IS* > 0.8, right side of Figure S2b, Supporting Information) for RNA coding potentials. By mapping 37‐GC onto the c‐JUN 5′‐UTR mRNA, three nucleobase pairs (colored in RED, BLUE, and YELLOW) were found. The red‐colored and blue‐colored pairs were located in the identified GREEN region, while the yellow‐colored one was not. Therefore, it is interesting to compare the contributions of these three 37‐GC pairs to the coding potential prediction. As shown on the right side of Figure 6b, the coding potential score (CPS) for all possible mutated sequences (a total of nine sequences for each pair, described in the step 5 of the Methods section) was calculated, and a green line was used to provide the CPS of the original sequence. The larger the CPS differences between the original sequence and those nine mutated ones were, the greater contributions of the corresponding 37‐GC pairs made. As illustrated in the lower‐right corner of Figure 6b, there were significant differences between the CPSs of yellow‐colored 37‐GC pair and that of the red‐colored/blue‐colored ones, which further led to distinct contribution made by the yellow‐colored, red‐colored, and blue‐colored pairs. In other words, the 37‐GC pairs located within the identified region (red and blue) contributed much more compared with that outside the region (yellow). All in all, *IntRNA* could effectively detect the impacts of sequence variations on coding potential and the roles of certain segments in RNA structure. Therefore, *IntRNA* could also be considered as a powerful method/tool for revealing the relationship between RNA structure and function.

### 2.6 In‐Depth Interpretation of ncRNA Functional Potential by Molecular Example

To further evaluate *IntRNA*’s interpretability, SNORD44, a snoRNA known to guide site‐specific 2′‐O‐methylation on 18S rRNA was analyzed.^[^ ^58^, ^59^, ^60^ ^]^ As shown in Figure S6a (Supporting Information), structural and functional elements, including the conserved Box C and Box D motifs (highlighted in red), were focused. These motifs are essential for snoRNA stability, maturation, and snoRNP complex formation.^[^ ^61^ ^]^ A pairwise mutation analysis of the 61‐nt SNORD44 sequence revealed that nucleotides 9‐12 and 24‐29 contributed most significantly to binding potential, partially overlapping with the Box C and D' motifs (red color in Figure S6a, Supporting Information). As shown in Figure S6b (Supporting Information), these regions also had higher probability scores predicted by *IntRNA* compared to surrounding sequences. Furthermore, gap‐based features, such as 14‐AU and 18‐AU (IS > 0.6; Figure S4c, Supporting Information) were analyzed. As shown in Figure S6c,d (Supporting Information), red‐colored 14‐AU and 18‐AU pairs located within functional motifs showed greater changes in probability scores upon mutation than gray‐colored pairs outside these regions.

In a similar way, circHIPK3, a circular RNA derived from exon 2 of the HIPK3 was adapted to further evaluate the interpretability and generalizability of *IntRNA*.^[^ ^62^ ^]^ Previous studies have reported that miR‐605^[^ ^63^ ^]^ and miR‐124^[^ ^64^ ^]^ can bind within the first 80‐nucleotides regions of circHIPK3, as shown in Figure S7a (Supporting Information). A pairwise mutation analysis of this 81‐nt segment revealed that nucleotides 36‐42 and 46‐55 contributed most significantly to interaction potential, overlapping precisely with the predicted binding sites for miR‐605 and miR‐124. These regions also exhibited higher binding probability scores, as predicted by *IntRNA* (Figure S7b, Supporting Information), compared to adjacent non‐functional sequences. Gap‐based interaction features were further examined, including 5‐CA and 13‐AA, which demonstrated strong importance scores (IS > 0.6; Figure S5b, Supporting Information). As shown in Figure S7c,d (Supporting Information), red‐colored 5‐CA and 13‐AA pairs located within miRNA binding motifs showed markedly greater differences in probability scores upon mutation than gray‐colored counterparts outside these regions. Together, these results underscore *IntRNA*’s capability to accurately pinpoint structurally and functionally relevant motifs in non‐coding RNAs, particularly those mediating miRNA‐RNA interactions.

Building upon these capabilities, *IntRNA* provides a unified and interpretable framework with strong predictive performance across *RNA universe*. It is particularly well‐suited for applications such as genome‐wide RNA annotation, evolutionary transcriptomics, and the discovery of functionally relevant RNAs from bulk sequencing data.^[^ ^65^, ^66^, ^67^, ^68^ ^]^ Nevertheless, the method has certain limitations: it requires substantial computational resources for large‐scale feature extraction and some features may exhibit limited utility depending on the specific classification task. Overall, *IntRNA* offers a robust and extensible foundation for RNA annotation, with clear opportunities for future improvement in scalability, adaptive feature selection, and integrative analysis.

## 3 Conclusion

The interpretation of *RNA universe* and coding potential are long‐standing issues in modern RNA studies, and three crucial questions remain unanswered: a) how to detect and interpret the coding potential of RNA, b) how to annotate the sophisticated taxonomy of the sncRNAs and c) how to successfully distinguish between circular and linear lncRNAs. In this study, a multi‐channel deep learning framework *IntRNA*, was thus constructed to interpret *RNA universe* and coding potential. First, a large number of RNA encoding features were proposed, which dramatically enlarged the available feature space. Second, a method realizing image‐like representation of RNA sequences was developed to describe the intrinsic correlation among the encoding features generated above. Third, a dual‐path multi‐channel model was developed, which consistently performed well when comparing with existing methods in a series of benchmark studies. The interpretability of *IntRNA* was tested by a real‐world study, which identified the key feature determining coding potential.

## 4 Experimental Section

Two key modules were integrated into the latest *IntRNA*: 1) the image‐like representation module “*RNAImage*” and 2) the dual‐path multi‐channel prediction module “CNN‐based model”. In order to construct the *IntRNA* model, five critical steps were employed here, which included: preparing datasets for model construction and performance assessments, generating new encoding features for enlarging the existing feature space, developing novel strategy enabling the representation of RNA as image, building the CNN‐based prediction model for interpreting *RNA universe*, and the in‐depth interpretation of the RNA coding potential based on feature ranking.

### Step 1. Preparing Datasets for Model Construction and Performance Assessment

To construct the *IntRNA* model, a total of 788576 unique human RNAs and their sequences were first gathered from *ENCODE* ^[^ ^69^ ^]^ and *Refseq*.^[^ ^70^ ^]^ Then, three benchmarks were collected by literature review to assess the performances of studied methods in solving the three key problems described in Figure 1. As detailed in Table S2a (Supporting Information), the dataset adopted to assess the methods’ performances in the detection and interpretation of RNA coding potential^[^ ^14^ ^]^ had a total of 180233 RNAs (including 112726 mRNAs & 67507 ncRNAs) from five different species: *Homo sapiens*, *Mus musculus*, *Danio rerio*, *Drosophila melanogaster* & *Saccharomyces cerevisiae*. Particularly, the data from *Homo sapiens* were split by the original publication^[^ ^14^ ^]^ into the training (comprising 33360 mRNAs and 24163 ncRNAs) and the test (comprising 8557 mRNAs and 8241 ncRNAs) datasets. During the training process, the strategy of fivefold cross‐validation was applied, and the test dataset was further enriched by integrating the data from the remaining four species.

Furthermore, the dataset adopted to evaluate the methods’ performances in the annotation of the sophisticated taxonomy of sncRNAs^[^ ^24^ ^]^ had a total of 8920 RNAs (as detailed in Table S2b, Supporting Information), and the partition of this dataset into training, validation and test followed the exactly same way as that in the original publication.^[^ ^24^ ^]^ Additionally, the benchmark adopted to assess the methods’ performance in distinguishing between circular and linear lncRNAs^[^ ^28^ ^]^ contained a total of 51625 RNAs (comprising 31942 circular & 19683 linear lncRNAs), and the dataset partition into training, validation and test followed the same way as that in the original publication.^[^ ^28^ ^]^

### Step 2. Generating New Encoding Features for Enlarging Existing Feature Space

In this study, a large number of new encoding features were generated to extensively expand the feature space of RNA representation. As shown in Figure 2, there were two types: the gap‐based features (*G‐features* containing a total of 724 newly‐proposed features) and encoding descriptors (*D‐features* comprising a total of 753 features and including 439 newly‐proposed ones). For both types, their containing sub‐groups (17 & 14 sub‐groups for *G‐features* & *D‐features*, respectively) were shown in the outer‐most layer, and the 14 sub‐groups of *D‐feature* were further summarized to three classes (sequence‐intrinsic, structure‐based & physicochemical property‐based). Those numbers in the brackets indicated the total numbers of features in the feature subgroups.

The *G‐feature* emphasized the distribution of nucleotides within RNA sequence, whereas the *D‐feature* gave the sequence‐intrinsic, structure‐based & physicochemical property‐based property of RNAs. As shown in Figure 3a, the way used in this study to generate *G‐features* was provided by taking the pairs of *G‐C* and *C‐G* as example, which were calculated by counting the frequency of nucleotide pairs at various intervals (from 0 to 40). For instance, *2‐GC* stood for the frequency of the nucleotide pair *G‐C* separated by two nucleotides within an RNA sequence, which resulted in a value of 1 for *2‐GC* in the example shown on the left side of Figure 3a. Similarly, the values resulted for *2‐CG*, *0‐CG*, *0‐GC*, *1‐GC*, and *1‐CG* equaled to 1, 2, 1, 1, and 1, respectively.

Furthermore, a total of 753 *D‐features* were generated in this study to encode the RNA sequences, 314 out of which were the classical encoding features (such as open reading frame, codon related, and secondary structures) proposed by previous reports,^[^ ^71^, ^72^, ^73^, ^74^, ^75^, ^76^, ^77^ ^]^ described in the Method S1 (Supporting Information). For the remaining 439 features, they were newly‐generated in this study based on specific set of physicochemical/structural properties (such as hydrogen bond basicity, topological polar surface area, and atom‐specific path lengths). Particularly, 439 *D‐features* were calculated using the strategy of Composition‐Transition‐Distribution (CTD). The detailed calculating method was systematically described in the Method S2 (Supporting Information). All in all, a total of 1477 encoding features were generated in this study to realize RNA representations, which included 724 *G‐features* and 753 *D‐features*. As illustrated on the right side of Figure 3a, a large number of encoding features/descriptors were generated in this study, which dramatically expanded (to over four times) the traditionally available feature space that encoded RNAs.

The feature sparsity was evaluated by calculating the fraction of zero values per feature across all samples in each task. As shown in Figure S8 (Supporting Information), most features exhibit low sparsity, indicating broad coverage and informative signal. To assess redundancy, pairwise cosine similarity analysis of *D‐* and *G‐features* was conducted. As shown in Figure S9 (Supporting Information), while most features are distinct, moderate similarity is observed among subsets derived from related physicochemical or structural properties.

### Step 3. Developing Novel Strategy Enabling the Representation of RNA as Image

A strategy titled *RNAImage* was further proposed to realize the image‐like representation of RNA for capturing the intrinsic correlations among the massive amount of RNA encoding features. As offered in Figure 3b, two template images (*D‐template* & *G‐template*) were first generated using a total of 788576 RNA sequences from two established knowledge bases (*ENCODE* and *RefSeq*) by following four sequential processes (from matrix generation^[^ ^78^ ^]^ to similarity calculation,^[^ ^79^ ^]^ then to dimension reduction,^[^ ^80^ ^]^ and finally to grid assignment^[^ ^81^ ^]^), which resulted in the template images of 28 × 27 (*D‐template*) and 27 × 27 (*G‐template*). Second, each analyzed RNA sequence could be converted to two “images” by mapping their intensities of encoding features to the corresponding locations in those two newly‐constructed template images based on our *RNAImage* strategy.

A detailed description on the application of *RNAImage* strategy was provided in Figure S10 (Supporting Information). First, each RNA sequence was converted into two feature‐matrices of distinct matrix dimension (753 × 788576 and 724 × 788576 for *D‐features* and *G‐features*, respectively). Second, each feature‐matrix was transformed to a similarity‐matrix by calculating the Cosine Similarities among studied features, which led to two similarity‐matrices of distinct dimension (753 × 753 and 724 × 724 for *D‐features* and *G‐features*, respectively). Third, UMAP approach was then adopted to reduce the dimension of each similarity‐matrix, and *J‐V algorithm* was further applied to each UMAP plot, which finally generated two template images of 28 × 27 (*D‐template*) and 27 × 27 (*G‐template*) dimension. Finally, each RNA sequence could be converted to two images by mapping their intensities of encoding features to the corresponding locations in two template images.

### Step 4. Building the CNN‐Based Prediction Model for Interpreting *RNA Universe*

A dual‐path multi‐channel model was constructed to facilitate the interpretation of *RNA universe*. As shown in Figure 3c, each RNA sequence was first converted to two images of different matrix dimension (28 × 27 & 27 × 27 for *D‐features* & *G‐features*, respectively) using *RNAImage* strategy described in step 3. Second, both images representing the studied RNA were then split to multiple channels according to the number of feature sub‐groups provided in Figure 2, which resulted in 17 and 14 channels for *D‐template* and *G‐template*, respectively. Third, those channels were then fed into a convolutional layer containing a single filter in a dual path manner, and a convolutional block was separately adopted to extract information (including a max‐pool layer, a convolutional layer with three filters and a concatenation layer), which was repeated twice to extract embedded features and resulted in a total of 192 matrices of 10 × 10 dimension in each path. Fourth, a global max pooling layer was separately applied to each path for converting the 192 matrices to a vector, and the vectors of two paths were then concatenated to generate new vector of 384 length. Finally, this newly‐generated vector was forwarded to a fully connected layer for RNA classification.

The classifiers were implemented using *Python TensorFlow* (v2.3.0) and Keras libraries (v2.4.3). The model employed categorical crossentropy as the loss function to measure the discrepancy between predicted class probabilities and true labels. For each of the three tasks (binary classification of RNA coding potential, multi‐class annotation of sncRNA taxonomy, and binary classification of circular vs linear lncRNAs), the final output layer of the model was adjusted accordingly. To assess the performances of *IntRNA* and available methods, six popular metrics were employed, which are specificity (SPE), sensitivity (SEN), precision (PRE), accuracy (ACC), area under the curve (AUC), and Matthews correlation coefficient (MCC). A grid search methodology was used to identify the optimal combination of hyperparameters (learning rate and batch size) in terms of the parameter tuning. Hyperparameter optimization for *IntRNA* was performed using MCC value. The batch size varied at 32, 64 and 96, and the learning rate was tested at 0.00001, 0.0001, 0.0002, and 0.0005. As provided in Figure S11 (Supporting Information), the *IntRNA* model exhibited insensitivity to both studied hyperparameters, as evidenced by the line charts providing MCC values. Training and validation accuracy and loss curves during training were provided in Figure S12 (Supporting Information) and parameter tuning of UMAP and dimensionality reduction method comparison were shown in Figure S13 (Supporting Information).

### Step 5. In‐Depth Interpretation of the RNA Coding Potential by Feature Ranking

To quantify the contribution of the studied RNA encoding features to the newly‐developed model, the importance score of each feature was measured by permutation algorithm.^[^ ^82^ ^]^ Particularly, the optimized cross‐entropy loss (OCEL) of the constructed model was first calculated using the data (provided in Table S2a, Supporting Information) applied to train the model for detecting and interpreting RNA coding potential. Second, permutations were applied to each feature on RNAs’ image‐like representations, and a permuted cross‐entropy loss (PCEL) was calculated for each feature. Third, the importance score (*IS*) of a studied feature was determined by the difference between its PCEL and OCEL. The higher the IS was, the greater the contribution of the feature to the *IntRNA* model was. This process above was iterated for all features until their ISs were fully obtained.

To measure the contribution of certain *G‐feature* in detecting coding potential, pairwise mutation analysis was further applied to confirm the contribution of an identified nucleotide pair to *IntRNA* model. As provided in the Figure S14 (Supporting Information), the nucleotide *G* and *C* were fully mutated, which resulted in a total of nine mutated sequences. The probability scores generated by the fully connected layers of the *IntRNA* model provided in Figure 3c were used to quantitatively measure the coding potential of all those ten sequences (including nine mutated and one original), and the average probability score of those nine mutated sequences could thus be represented as:

(1) $$P_{mutated}=\frac{1}{9}\times \sum_{i=1}^{3}\sum_{j=1}^{3}P_{i,j}$$

where *P_i,j_* indicated the probability score of the sequences (with mutations at the studied positions) predicted by *IntRNA* model. As a result, the contributions of the studied nucleotide pair to *IntRNA* model could therefore be described using the following Equation (2):

(2) $$C_{nucleotidepair}=P_{original}-P_{mutated}$$

The higher the *C_nucleotide pair_* was, the greater contribution the studied pair made to coding potential. For visualizing RNA structure, the *RNArtist* (https://github.com/fjossinet/RNArtist) was adopted in this study to convert 3D RNA structure into a 2D plot.

### Statistical Analysis

In this work, the statistical tests and the definition of *p*‐values are provided in the figure legends. Statistical analysis was performed in R language.

## Conflict of Interest

The authors declare no conflict of interest.

## Author Contributions

Y.X.W., M.J.M., and S.J.H. contributed equally to this work as co‐first authors. F.Z. conceived the idea and designed the research. Y.X.W., M.J.M., S.J.H., and Q.X.Y. developed the model and debug source codes. Y.X.W., M.J.M., S.J.H., and Q.X.Y. performed the benchmark data analyses. Y.X.W., M.J.M., S.J.H., W.Z., Z.Q.P., J.T., and Q.X.Y. contributed to statistics and data visualization. Y.X.W., M.J.M., Q.X.Y., and S.J.H. constructed the online server. F.Z., Q.X.Y., Y.X.W., and S.J.H. wrote the manuscript. All authors provided critical feedback to the research.

## Supporting information



Supporting Information

## Data Availability

The source codes and datasets are accessible at: https://github.com/idrblab/intrna.
