# Supplementary material for: Interpretation of RNA Universe and Coding Potential Using IntRNA
Source: Adv Sci (Weinh). 2025 Aug 22;12(43):e09518. doi: 10.1002/advs.202509518 (PMC12631819; doi:10.1002/advs.202509518)
Supplement: Supplementary file 1 — Supporting Information [file ADVS-12-e09518-s001.pdf]

Supplementary Information for:

Interpretation of RNA Universe and Coding Potential Using *IntRNA*

Yunxia Wang[1,2,†], Minjie Mou[1,†], Shijie Huang[1,†], Wei Zhang[1], Ziqi Pan[1], Jing Tang[3], Yihao Wang[4], Qingxia Yang[5,*], Feng Zhu[1,2,*]

[1] College of Pharmaceutical Sciences, The Second Affiliated Hospital, Zhejiang University School of Medicine, State Key Laboratory of Advanced Drug Delivery and Release Systems, Zhejiang University, Hangzhou 310058, China

[2] Innovation Institute for Artificial Intelligence in Medicine of Zhejiang University, Alibaba-Zhejiang University Joint Research Center of Future Digital Healthcare, Hangzhou 330110, China

[3] College of Basic Medicine, Chongqing Medical University, Chongqing 400016, China

[4] The High School Affiliated to Renmin University of China, No. 37 Zhongguancun Street. Haidian District, Beijing 100080, China

[5] Zhejiang Provincial Key Laboratory of Precision Diagnosis and Therapy for Major Gynecological Diseases, Women's Hospital, Zhejiang University School of Medicine, Hangzhou 310058, China

* Corresponding Authors: Feng Zhu (zhufeng@zju.edu.cn) and Qingxia Yang (yangqx@zju.edu.cn)

† These authors contributed equally to this work as co-first authors.

Table S1. Performance comparisons for solving three *key problems* among *IntRNA* and three available tools (*CPPred*, *circDeep*, and *nRC*). The best-performing value under each evaluating metric for those *key problems* was highlighted using BOLD and the metrics included: *Matthews correlation coefficient* (MCC), *specificity* (SPE), *sensitivity* (SEN), *precision* (PRE) & *accuracy* (ACC).

***a.* Detection and Interpretation of RNA Coding Potential**

Method	MCC	SPE	SEN	PRE	ACC
CPPred	0.926	0.972	0.954	0.973	0.963
circDeep	0.905	0.945	0.959	0.948	0.952
nRC	0.609	0.697	0.900	0.742	0.797
IntRNA	**0.942**	**0.983**	**0.960**	**0.983**	**0.971**

***b.* Annotation of the Sophisticated Taxonomy of sncRNAs**

Method	MCC	SPE	SEN	PRE	ACC
nRC	0.803	0.985	0.818	0.815	0.818
CPPred	0.746	0.980	0.765	0.765	0.765
circDeep	0.883	0.991	0.892	0.895	0.892
IntRNA	**0.906**	**0.993**	**0.913**	**0.914**	**0.913**

***c.* Distinguishing between Circular and Linear lncRNAs**

Method	MCC	SPE	SEN	PRE	ACC
circDeep	0.693	0.770	0.910	0.865	0.857
CPPred	0.810	0.858	**0.943**	0.915	0.911
nRC	0.480	0.869	0.587	0.733	0.761
IntRNA	**0.827**	**0.891**	0.936	**0.933**	**0.918**

Table S2. Three benchmark datasets collected for model construction and performance assessment in this study. These benchmarks were adopted to evaluate the performance of various methods in solving three *key problems* illustrated in **Figure 1** and described in the following section as table legends.

***a.* Detection and Interpretation of RNA Coding Potential** (*five-fold cross-validation*)

Dataset Split	Species Origin	No. of mRNAs	No. of ncRNAs
Data for Training	*Homo sapiens* (human)	33,360	24,163
Data for Test	*Homo sapiens* (human)	8,557	8,241
	Mus musculus (mouse)	31,102	19,930
	Danio rerio (zebrafish)	15,594	10,662
	Drosophila melanogaster (fruit fly)	17,400	4,098
	Saccharomyces cerevisiae (yeast)	6,713	413
	Arabidopsis thaliana (thale cress)	11,308	11,308

***b.* Annotation of the Sophisticated Taxonomy of sncRNAs** (*training-validation-test split*)

Dataset Split	No. of sncRNAs in Each of the 13 Sub-classes	Total No. of sncRNAs
Data for Training	400 in each sub-class (with 1 exception of 256)	5,056
Data for Validation	100 in each sub-class (with 1 exception of 64)	1,264
Data for Test	200 in each sub-class	2,600

***c.* Distinguishing between Circular and Linear lncRNAs** (*training-validation-test split*)

Dataset Split	Species Origin	No. of Circular	No. of Linear
Data for Training	*Homo sapiens* (human)	23,959	14,763
Data for Validation	*Homo sapiens* (human)	3,193	1,968
Data for Test	*Homo sapiens* (human)	4,790	2,952

Table S3. The evolutionary relations among five studied species: *Homo sapiens* (human), *Mus musculus* (mouse), *Danio rerio* (zebrafish), *Drosophila melanogaster* (fruit fly), and *Saccharomyces cerevisiae* (yeast), which were also illustrated in **Figure 4*b***.

	Kingdom	Phylum	Class	Order	Family	Genus	Species
**Human**	*Animalia*	*Chordata*	*Mammalia*	*Primates*	*Hominidae*	*Homo*	*H. sapiens*
**Mouse**	*Animalia*	*Chordata*	*Mammalia*	*Rodentia*	*Muridae*	*Mus*	*M. musculus*
**Zebrafish**	*Animalia*	*Chordata*	*Actinopterygii*	*Cypriniformes*	*Cyprinidae*	*Danio*	*D. rerio*
**Fruit fly**	*Animalia*	*Arthropoda*	*Insecta*	*Diptera*	*Drosophilidae*	*Drosophila*	*D. melanogaster*
**Yeast**	*Fungi*	*Ascomycota*	*Saccharomycetes*	*Saccharomycetales*	*Saccharomycetaceae*	*Saccharomyces*	*S. cerevisiae*

Table S4. Summary of representative computational methods for RNA annotation.

Function	Tool	Algorithm	Encoding Features	Feature class	Reference
RNA coding potential	CPAT	Logistic regression	ORF size, ORF coverage, Fickett score, hexamer usage bias	Sequence-intrinsic	*Nucleic Acids Res.* 41: e74, 2013
	PLEK	SVM	Improved k-mer	Nucleotide counts-based	*BMC Bioinformatics.* 15: 311, 2014
	CPPred	RF	ORF length, ORF coverage, Fickett score, Hexamer Score, CTD	Sequence-intrinsic	*Nucleic Acids Res.* 47: e43, 2019
	CPC2	SVM	Fickett score, ORF length, ORF integrity, isoelectric point, Gravy, Instability index	Physicochemical properties-directed	*Nucleic Acids Res.* 45: 12-16, 2017
	FEELnc	RF	ORF coverage, k-mer, RNA sequence length	Sequence-intrinsic	*Nucleic Acids Res.* 45: e57, 2017
	RNAmining	SVM + LightGBM	k-mer	Nucleotide counts-based	*F1000Research.* 10: 323, 2021
	DeepCPP	CNN	Nucleotide bias, discontinuous k-mer, hexamer score, ORF coverage, ORF length	Nucleotide counts-based	*Brief Bioinform.* 22: 2073-84, 2021
	CPE-SLDI	XGBoost	ORF length, ORF coverage, Fickett score, Hexamer score, ORF integrity, isoelectric point(pI), Gravy, Instability index, CTD	Physicochemical properties-directed	*IEEE/ACM Trans Comput Biol Bioinform.* 19: 1075-83, 2022
Sophisticated taxonomy of sncRNAs	nRC	CNN	Secondary structure	Structure-based	*BioData Min.* 10: 27, 2017
Circular and linear lncRNAs	circDeep	ACNN-BLSTM	RCM descriptor, Conservation descriptor, k-mer	Nucleotide counts-based	*Bioinformatics.* 36: 73-80, 2020

Table S5. Performance assessment among *IntRNA* and six existing methods for detecting RNA coding potential. The performances were evaluated by testing on five different species (the species taxonomy was described in **Figure 4*b***) and based on six measures: *specificity* (SPE), *sensitivity* (SEN), *precision* (PRE), *accuracy* (ACC), *area under the curve* (AUC), and *Matthews correlation coefficient* (MCC).

Dataset	Methods	AUC	MCC	SPE	SEN	PRE	ACC
Homo sapiens (human)	CPPred	0.987	0.894	0.954	0.940	0.955	0.947
	CPAT	0.983	0.884	0.946	0.940	0.956	0.943
	CPC2	0.981	0.863	0.951	0.912	0.951	0.931
	PLEK	0.992	0.932	0.979	0.954	0.981	0.966
	RNAmining	0.965	0.797	0.892	0.905	0.897	0.899
	CPE-SLDI	0.957	0.680	0.982	0.661	0.975	0.822
	IntRNA	**0.996**	**0.942**	0.983	0.960	0.983	0.970
Mus musculus (mouse)	CPPred	0.993	0.927	0.977	0.956	0.985	0.964
	CPAT	0.988	0.906	0.954	0.961	0.979	0.959
	CPC2	0.990	0.909	0.957	0.956	0.972	0.956
	PLEK	0.956	0.751	0.906	0.876	0.954	0.886
	RNAmining	0.962	0.778	0.906	0.882	0.936	0.891
	CPE-SLDI	0.994	0.931	0.976	0.960	0.984	0.967
	IntRNA	**0.995**	**0.942**	0.979	0.967	0.986	0.972
Danio rerio (zebrafish)	CPPred	0.980	0.900	0.932	0.966	0.954	0.952
	CPAT	0.949	0.805	0.761	0.986	0.908	0.920
	CPC2	0.970	0.869	0.898	0.964	0.932	0.937
	PLEK	0.941	0.718	0.821	0.905	0.920	0.879
	RNAmining	0.944	0.745	0.852	0.894	0.898	0.877
	CPE-SLDI	0.966	0.883	0.901	0.955	0.927	0.933
	IntRNA	**0.985**	**0.902**	0.915	0.978	0.944	0.953
Drosophila melanogaster (fruit fly)	CPPred	0.987	0.863	0.925	0.964	0.982	0.956
	CPAT	0.989	0.888	0.949	0.976	0.991	0.973
	CPC2	0.989	0.894	0.944	0.972	0.987	0.966
	PLEK	0.927	0.552	0.875	0.832	0.977	0.838
	RNAmining	0.930	0.659	0.755	0.923	0.941	0.891
	CPE-SLDI	0.990	0.884	0.967	0.960	0.992	0.961
	IntRNA	**0.993**	**0.915**	0.960	0.976	0.990	0.973
Saccharomyces cerevisiae (yeast)	CPPred	0.990	0.506	1.000	0.856	1.000	0.864
	CPAT	0.967	0.219	1.000	0.872	1.000	0.873
	CPC2	0.983	0.561	1.000	0.888	1.000	0.894
	PLEK	0.803	0.067	0.913	0.486	0.999	0.489
	RNAmining	0.958	0.285	0.985	0.616	0.999	0.638
	CPE-SLDI	0.995	0.514	1.000	0.861	1.000	0.869
	IntRNA	**0.997**	**0.664**	1.000	0.931	1.000	0.935

Table S6. List of the top 21 D-features and G-features identified by feature importance ranking, along with their corresponding subgroups.

D-features				
**Rank**	**Features**	**Feature Subgroup**	**Importance score in training dataset**	**Importance score in test dataset**
1	EiPS0: Electron-ion interaction pseudopotential spectrum 0	EIIP based spectrum	0.1506	0.1194
2	ORFCo: ORF coverage	Open reading frame	0.1161	0.0954
3	ORFIn: ORF integrity	Open reading frame	0.1092	0.0840
4	APTW1: Dinucleotide auto covariance of lag1 on PTW	Nucleotide related	0.0498	0.0379
5	CNHB4: NH- count distribution of 1.00B	Molecular fingerprint	0.0350	0.0426
6	DPRT4: Distribution of 1.00T	Transcript related	0.0247	0.0290
7	DPRT3: Distribution of 0.75T	Transcript related	0.0232	0.0170
8	DPRG3: Distribution of 0.75G	Transcript related	0.0194	0.0122
9	SHAA4: Strong H-Bond acceptors_3 distribution of 1.00A	Hydrogen bond related	0.0173	0.0192
10	EiPS1: Electron-ion interaction pseudopotential spectrum 0.25	EIIP based spectrum	0.0163	0.0102
11	DPRA3: Distribution of 0.75A	Transcript related	0.0162	0.0117
12	MLFB4: Linear free energy distribution of 1.00B	Hydrogen bond related	0.0148	0.0162
13	HPCB4: Gas-hexadecane PC_3 distribution of 1.00B	Partition coefficient	0.0129	0.0139

14	SHAB3: Strong H-Bond acceptors_3 distribution of 0.75B	Hydrogen bond related	0.0111	0.0075
15	EipSP: Electron-ion interaction pseudopotential signal peak	EIIP based spectrum	0.0111	0.0063
16	DPRC3: Distribution of 0.75C	Transcript related	0.0108	0.0065
17	PHBB4: Potential Hydrogen Bonds_3 distribution of 1.00B	Hydrogen bond related	0.0102	0.0109
18	SLFA4: Sum of path lengths starting from oxygens_3 distribution of 1.00A	Topological indice	0.0098	0.0104
19	DPRA4: Distribution of 1.00A	Transcript related	0.0095	0.0068
20	LFIB3: Lipoaffinity index distribution of 0.75B	Solubility lipoaffinity	0.0090	0.0061
21	LFIB4: Lipoaffinity in.dex distribution of 1.00B	Solubility lipoaffinity	0.0088	0.0058
		**G-features**		
**Rank**	**Features**	**Feature Subgroup**	**Importance score in training dataset**	**Importance score in test dataset**
1	A	1,3 mer	0.0556	0.0403
2	35-UU	Gap feature U-U	0.0522	0.0353
3	26-UU	Gap feature U-U	0.0399	0.0268
4	39-GA	Gap feature G-A	0.0375	0.0262
5	35-GG	Gap feature G-G	0.0372	0.0264

6	U	1,3 mer	0.0358	0.0427
7	20-UU	Gap feature U-U	0.0346	0.0229
8	KMAGA: Transcript k-mer AGA content	1,3 mer	0.0289	0.0196
9	14-UU	Gap feature U-U	0.0257	0.0165
10	38-GG	Gap feature G-G	0.0257	0.0176
11	29-GG	Gap feature G-G	0.0250	0.0165
12	40-GU	Gap feature G-U	0.0234	0.0155
13	28-CA	Gap feature C-A	0.0224	0.0147
14	23-GG	Gap feature G-G	0.0211	0.0132
15	32-AA	Gap feature A-A	0.0202	0.0134
16	40-CA	Gap feature C-A	0.0194	0.0130
17	12-CA	Gap feature C-A	0.0193	0.0127
18	1-CA	Gap feature C-A	0.0189	0.0126
19	37-UA	Gap feature U-A	0.0188	0.0119
20	37-GC	Gap feature G-C	0.0186	0.0120
21	38-UU	Gap feature U-U	0.0180	0.0116

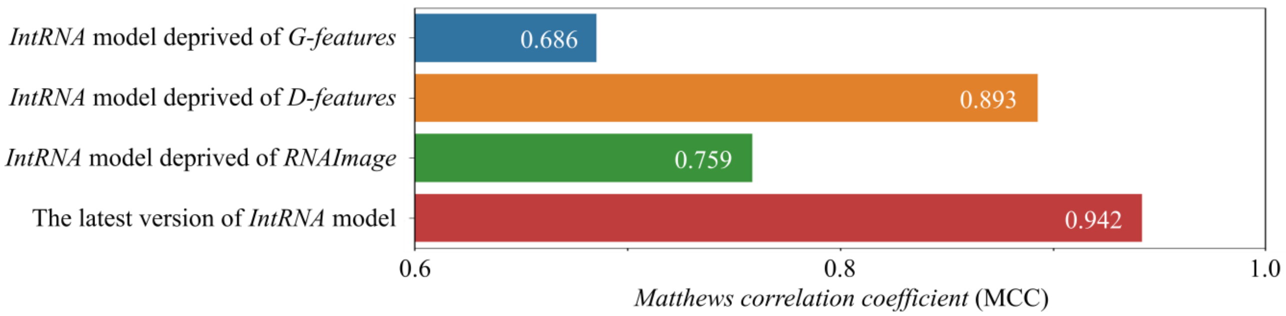

Figure S1. The results of *ablation study* for the constructed *IntRNA* model.

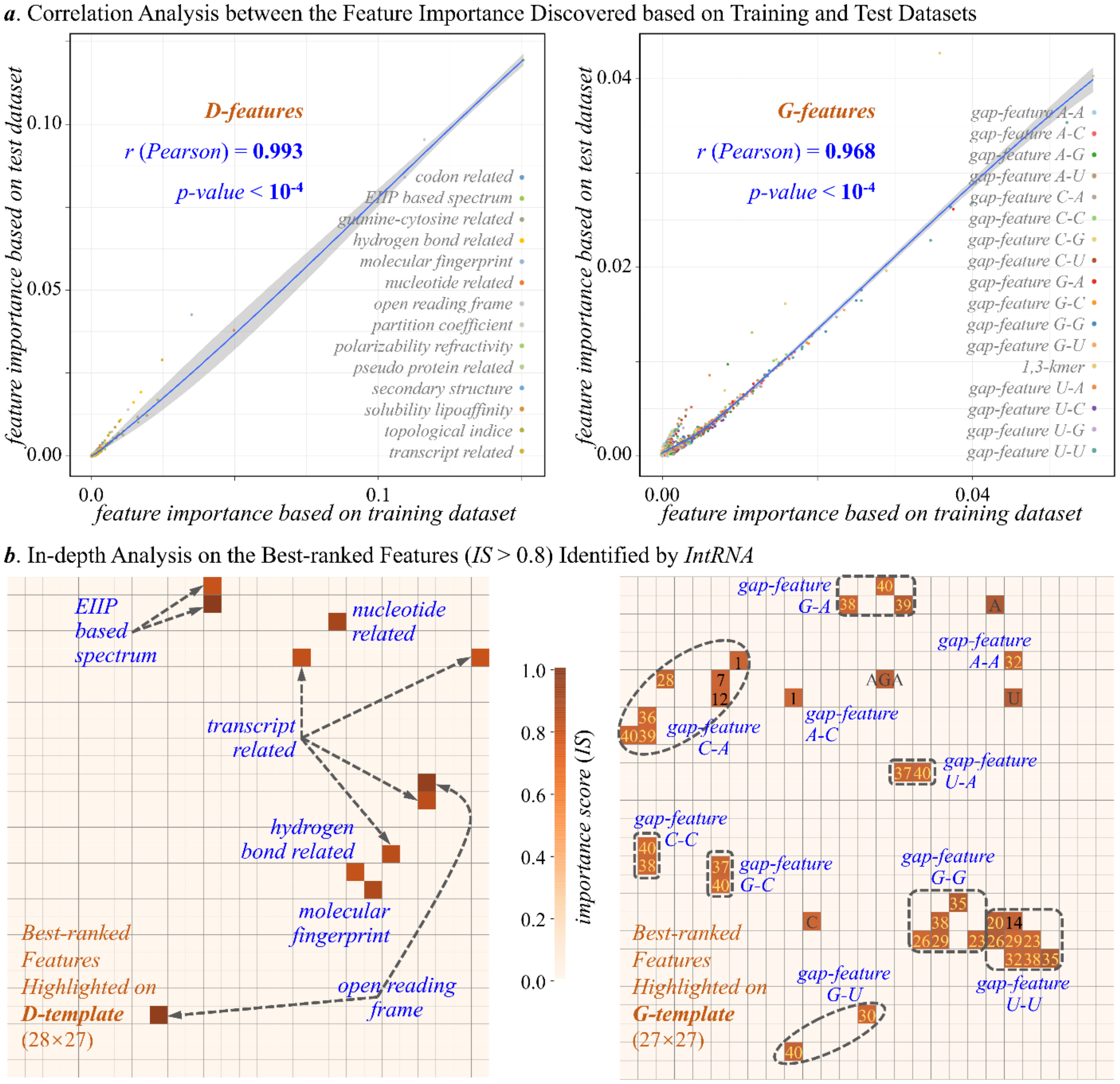

Figure S2. The in-depth analyses of those features identified in the *key problem* of detecting and interpreting the coding potential of RNA. (***a***) correlation analysis between the feature importance discovered based on training and test datasets. Particularly, the correlations in feature importance for *D-features* and *G-features* were discovered, which led to the significant *Pearson* correlations equaling to 0.993 and 0.968, respectively. (***b***) in-depth analyses on best-ranked features (*IS* > 0.8) identified by *IntRNA*. The exact locations of the best-ranked features on *RNAImage* template (*D-template* and *G-template* shown in **Figure 3*b***) and the feature subgroups (colored in BLUE) were described. The number in each square denoted the gap (*No.* of nucleobases) between two studied nucleobases (< 20 and ≥ 20 were highlighted in BLACK and YELLOW, respectively).

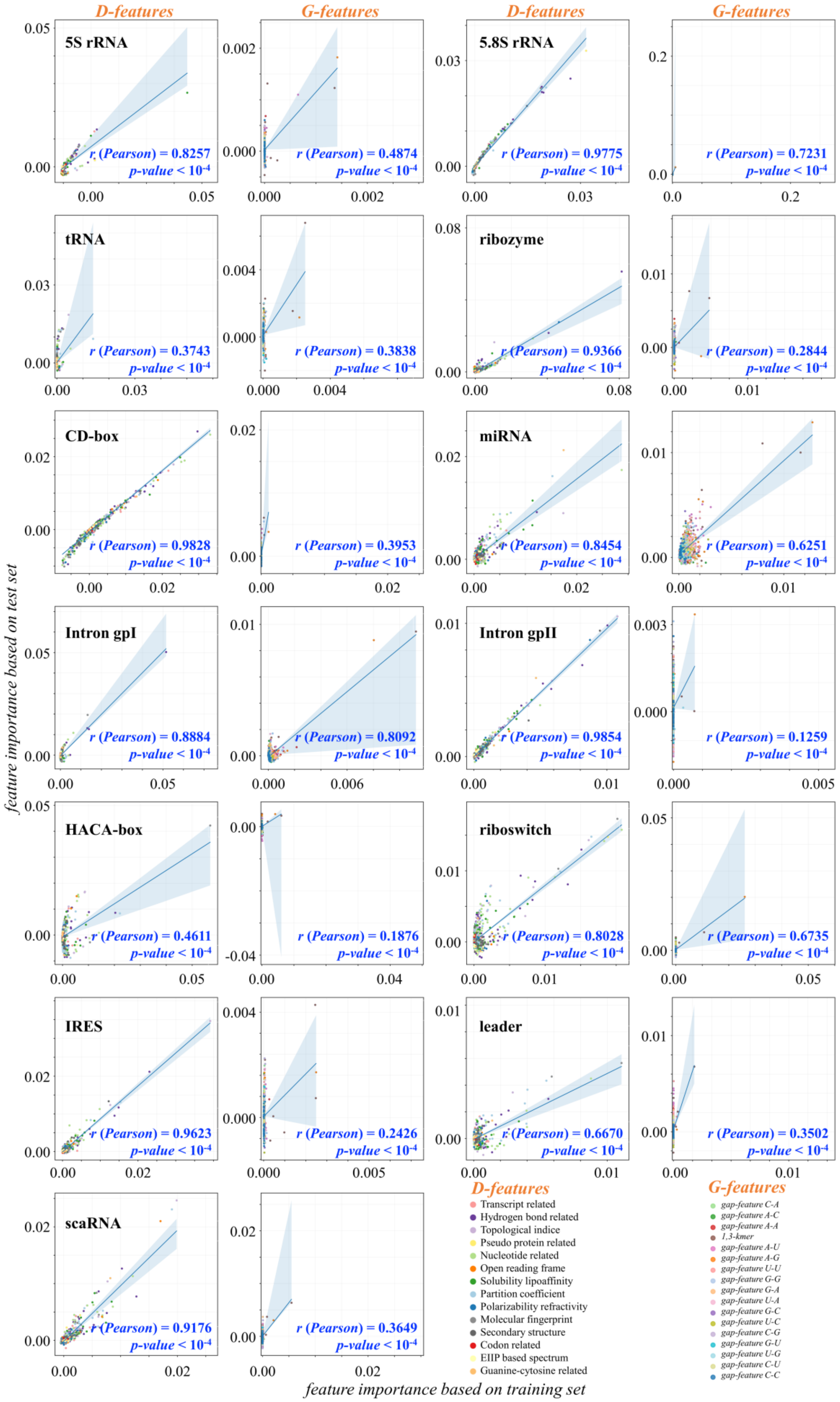

Figure S3. Correlation analysis between the feature importance discovered based on training and test datasets for D-features and G-features, evaluated across 13 classes of non-coding RNAs.

a. In-depth Analysis on Best-ranked Features (*IS* > 0.6) Identified *IntRNA*

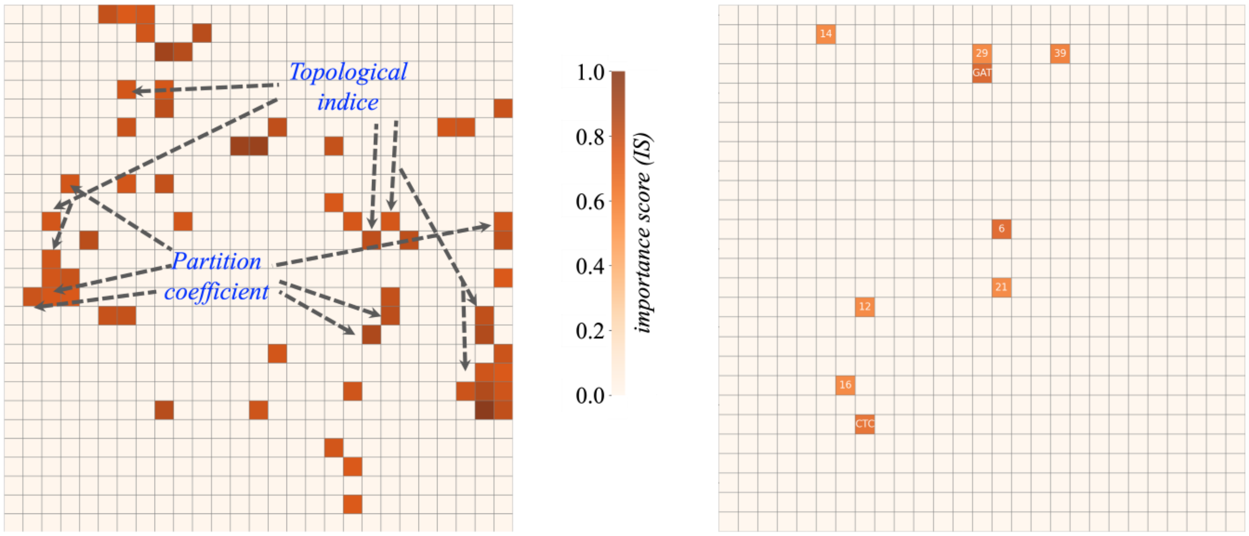

b. In-depth Analysis on Best-ranked Features (*IS* > 0.6) Identified *IntRNA*

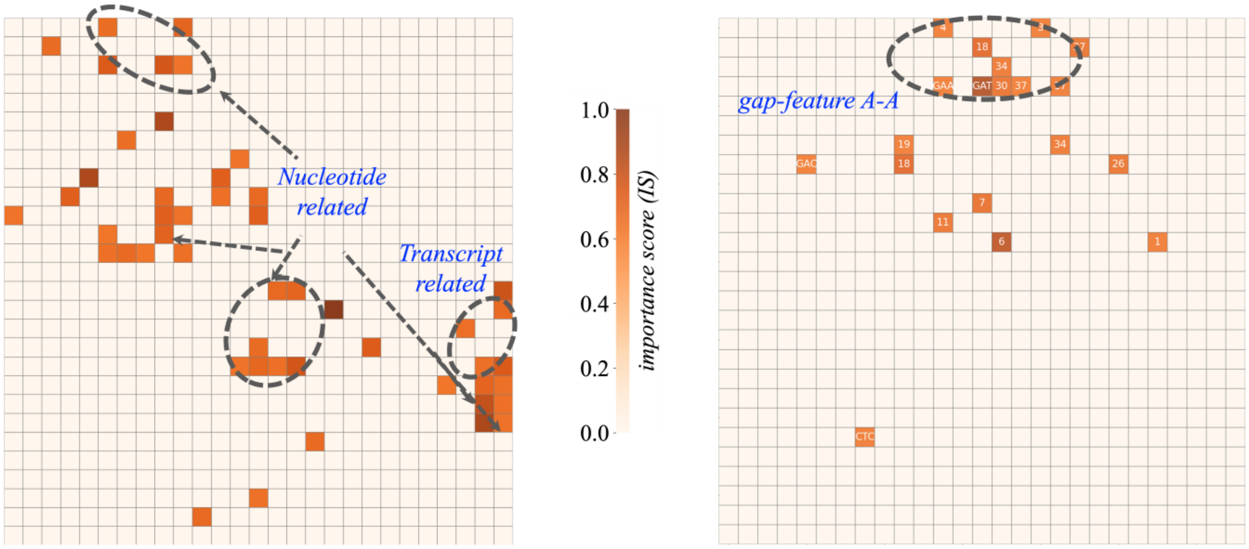

c. In-depth Analysis on Best-ranked Features (*IS* > 0.6) Identified *IntRNA*

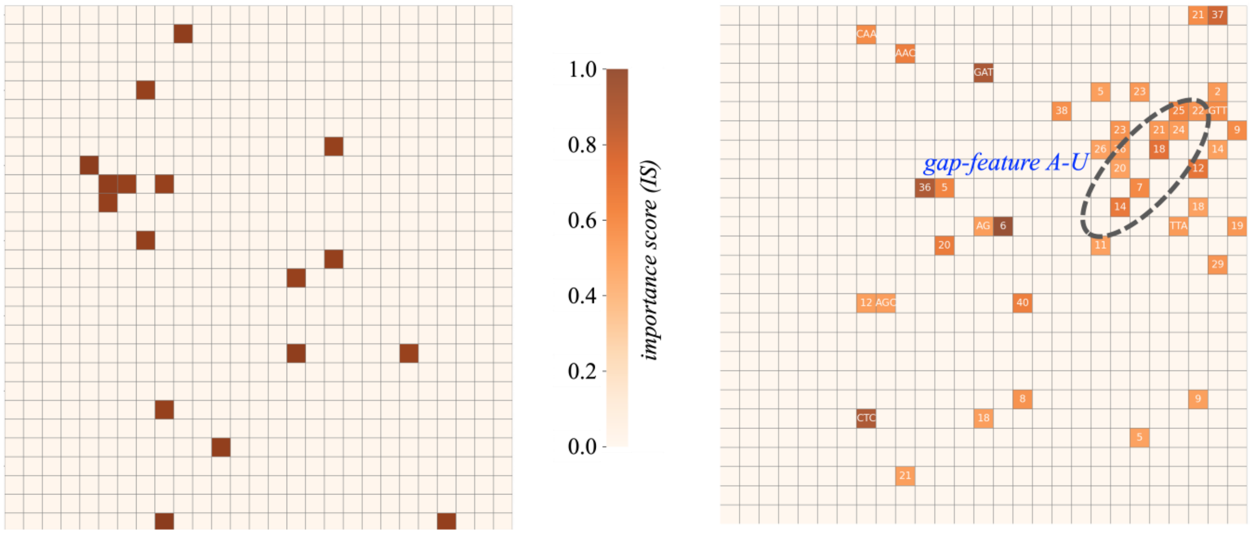

Figure S4. The in-depth analyses of those features identified in the *key problem* of detecting and interpreting the annotation of ncRNA. In-depth analyses on best-ranked features (*IS* > 0.6) identified by *IntRNA* for (***a***) the ribozyme RNAs, (***b***) Intron and (***c***) CD-box RNA. The exact locations of the

best-ranked features on *RNAImage* template (*D-template* and *G-template* shown in **Figure 3*b***) and the feature subgroups (colored in BLUE) were described. The number in each square denoted the gap (*No.* of nucleobases) between two studied nucleobases.

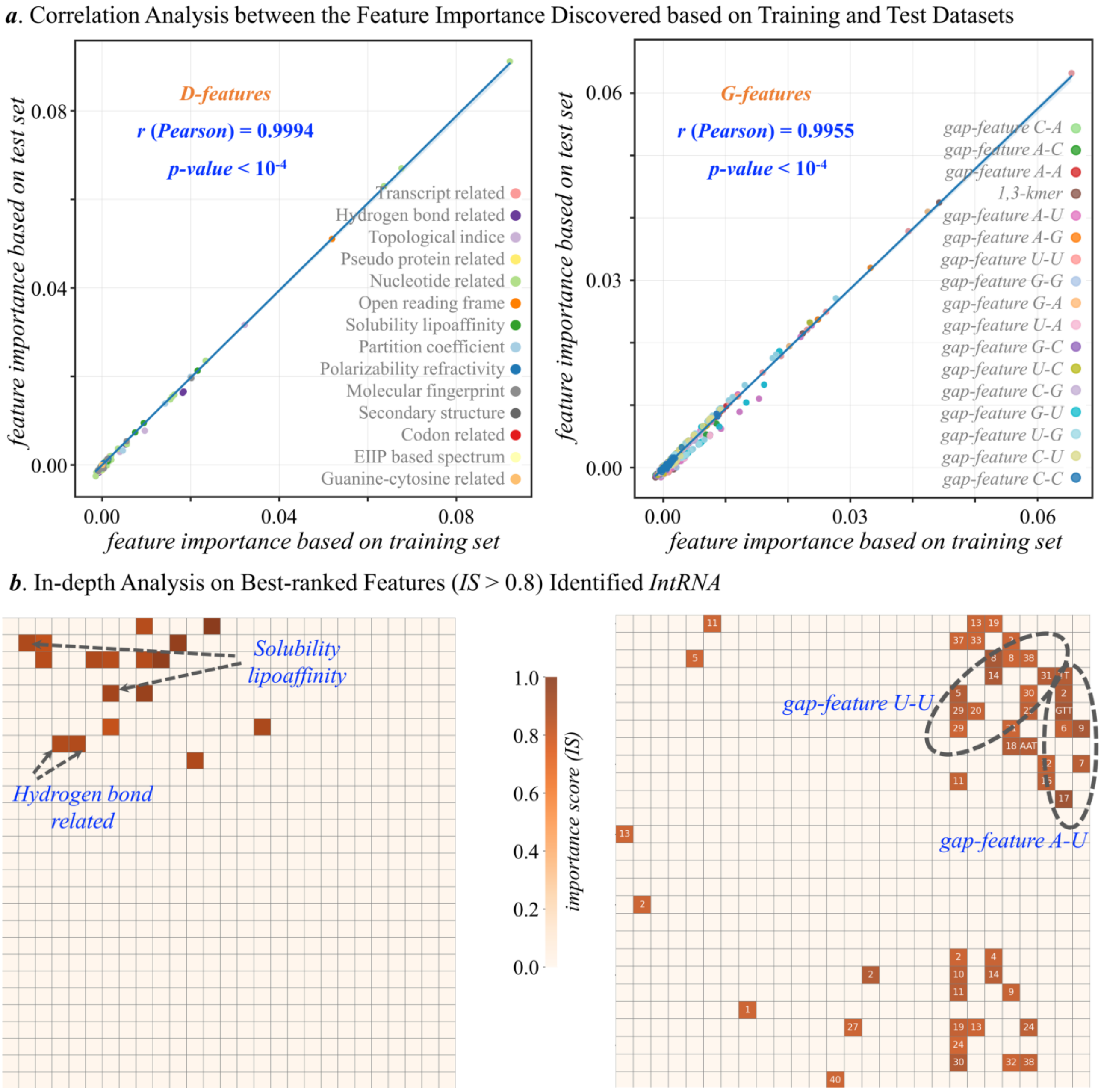

Figure S5. The in-depth analyses of those features identified in the *key problem* of detecting and interpreting the circRNA and linear RNA. (***a***) correlation analysis between the feature importance discovered based on training and test datasets. Particularly, the correlations in feature importance for *D-features* and *G-features* were discovered, which led to the significant *Pearson* correlations equaling to 0.9994 and 0.9955, respectively. (***b***) in-depth analyses on best-ranked features (*IS* > 0.8) identified by *IntRNA*. The exact locations of the best-ranked features on *RNAImage* template (*D-template* and *G-template* shown in **Figure 3*b***) and the feature subgroups (colored in BLUE) were described. The number in each square denoted the gap (*No.* of nucleobases) between two studied nucleobases.

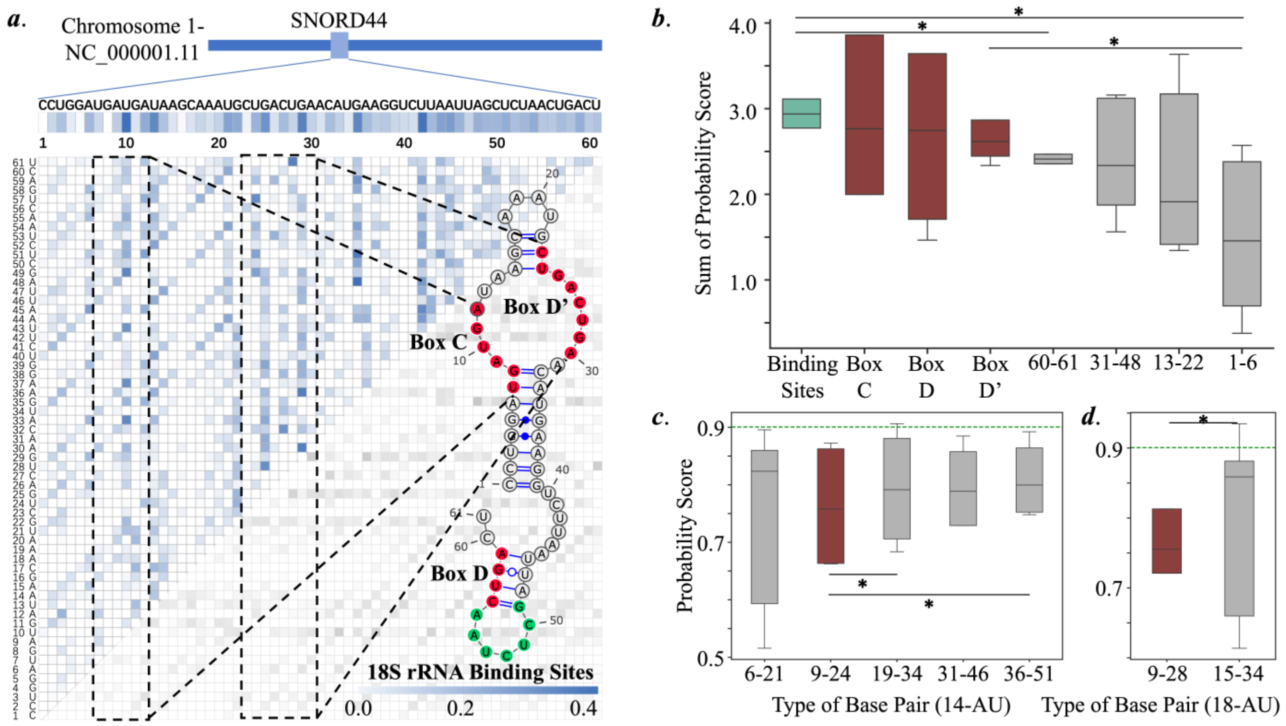

Figure S6. Interpretation of ncRNA binding ability by taking CD-box RNA SNORD44 as an example. (***a***) The pairwise mutation analyses of SNORD44 (containing 61 nucleotides) reveals functional structural elements (Box C, Box D and 18S rRNA-binding sites). Box C and Box D' are clearly highlighted with red color as structurally conserved motifs in the upper hairpin, while Box D is in the lower stem-loop and 18S rRNA-binding sites are located in the lower stem-loop. (***b***) Boxplots illustrate the effects of mutating specific structural elements on predicted functional probability scores. The red and green-colored boxplots represent key structural elements (Box C, Box D and 18S rRNA-binding sites), with comparisons to other structural elements shown in gray. (***c***) 14-AU were found at five different positions (6-21, 9-24, 19-34, 31-46 and 36-51) in SNORD44 (as shown in the lower structure of (***a***)), and boxplots of functional probability scores were applied for visualizing the importance of 14-AU found at different locations. (***d***) 18-AU were found at two different positions (9-28 and 15-34) in SNORD44 (as shown in the lower structure of (***a***)), and boxplot of functional probability scores were applied for visualizing the importance of 18-AU found at different locations. The green dashed line in (***c***) and (***d***) showed the functional probability scores of the original SNORD44 sequence. The *p*-value <0.05 was denoted using one asterisks (*).

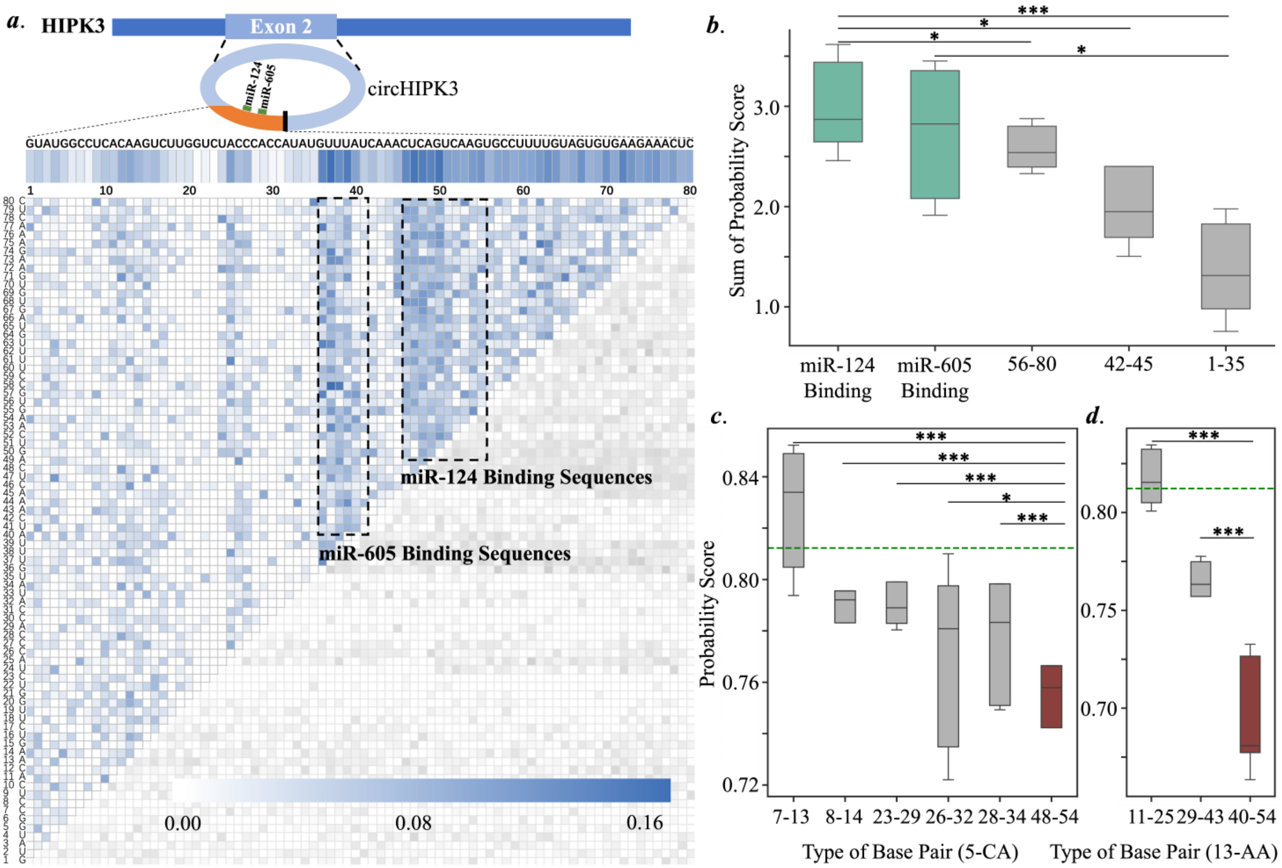

Figure S7. Interpretation of circRNA binding ability by taking circHIPK3 (has-circ-0000284) as an example. (***a***) The pairwise mutation analyses of circHIPK3 segment (containing 80 nucleotides) reveals miR-605 and miR-124 binding sites. (***b***) Boxplots on the right illustrate the effects of mutating specific structural elements on predicted functional probability scores. The green-colored boxplots represent key structural elements (miR-605 and miR-124 binding sites), with comparisons to other structural elements shown in gray. (***c***) 5-CA were found at six different positions (7-13, 8-14, 23-29, 26-32, 28-34 and 48-54) in circHIPK3 segment and boxplots of functional probability scores were applied for visualizing the importance of 5-CA found at different locations. (***d***) 13-AA were found at three different positions (11-25, 29-43 and 40-54) in circHIPK3 segment and a boxplot of functional probability scores were applied for visualizing the importance of 13-AA found at different locations. The green dashed line in (***c***) and (***d***) showed the functional probability scores of the original circHIPK3 sequence. The *p*-value <0.05 was denoted using two asterisks (*), while the *p*-value <0.001 were indicated using three asterisks (***).

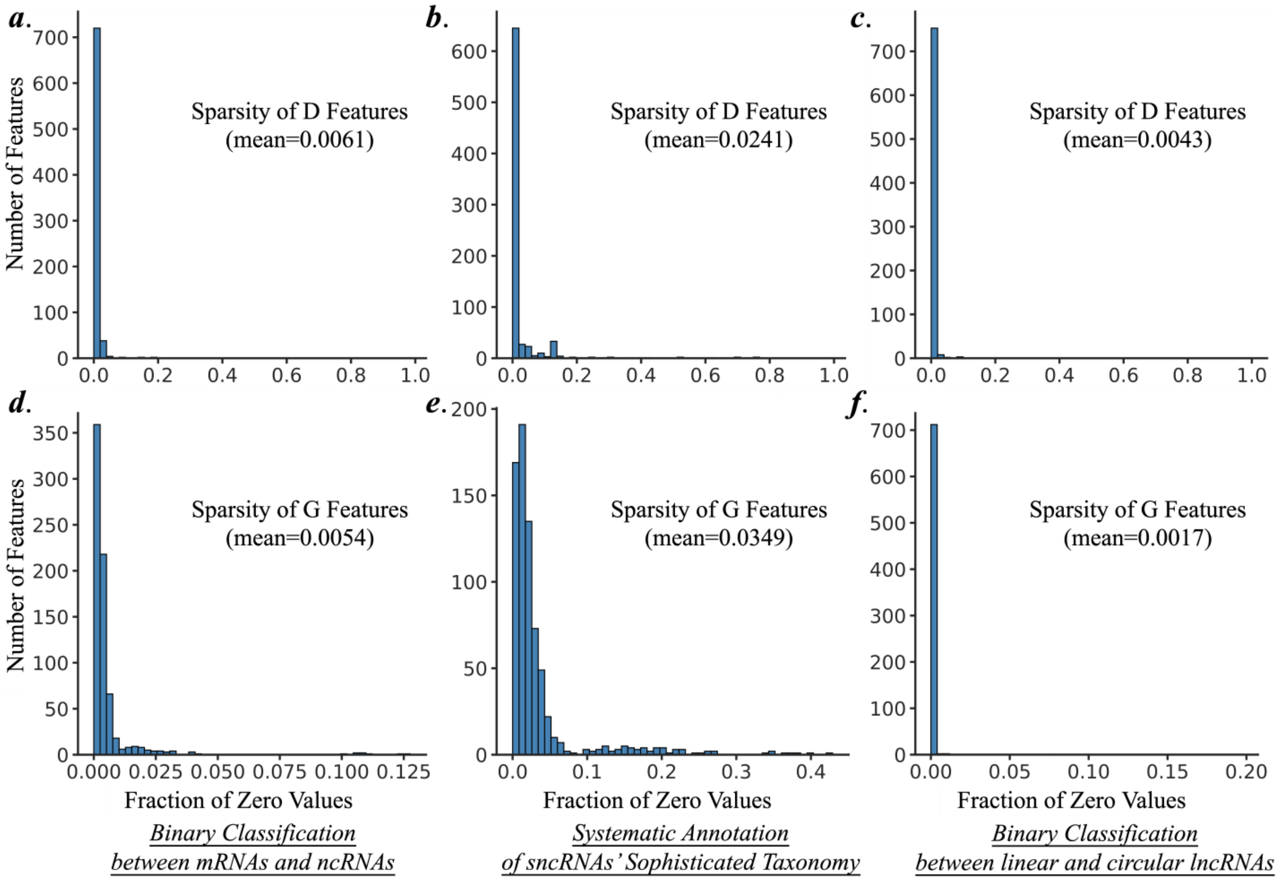

Figure S8. Distribution of zero fractions for D- and G-features across three key problems: how to detect and interpret RNA coding potential in ***a***) and ***d***); how to annotate the sophisticated taxonomy of sncRNAs in ***b***) and ***e***); and how to successfully distinguish between circular and linear lncRNAs in ***c***) and ***f***). The "mean" represents the average fraction of zeros for all feature across all samples within the given classification task.

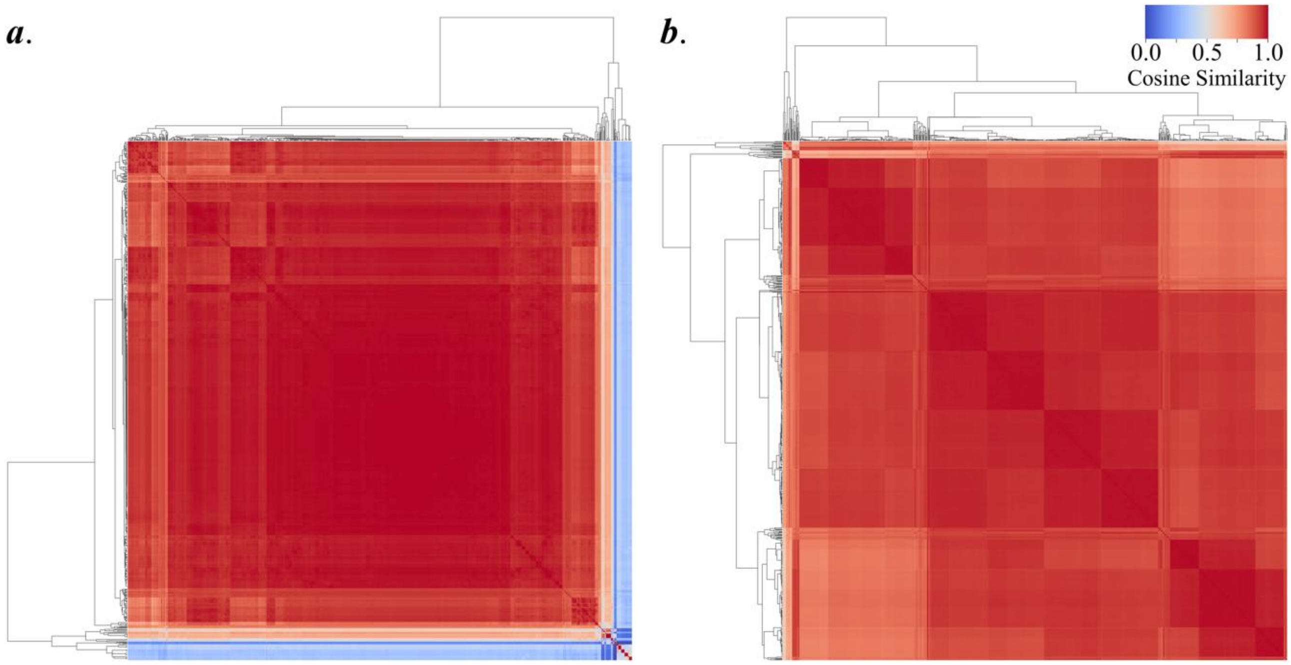

Figure S9. Cosine similarity of fearers in two template images: (***a***) D-template & (***b***) G-template.

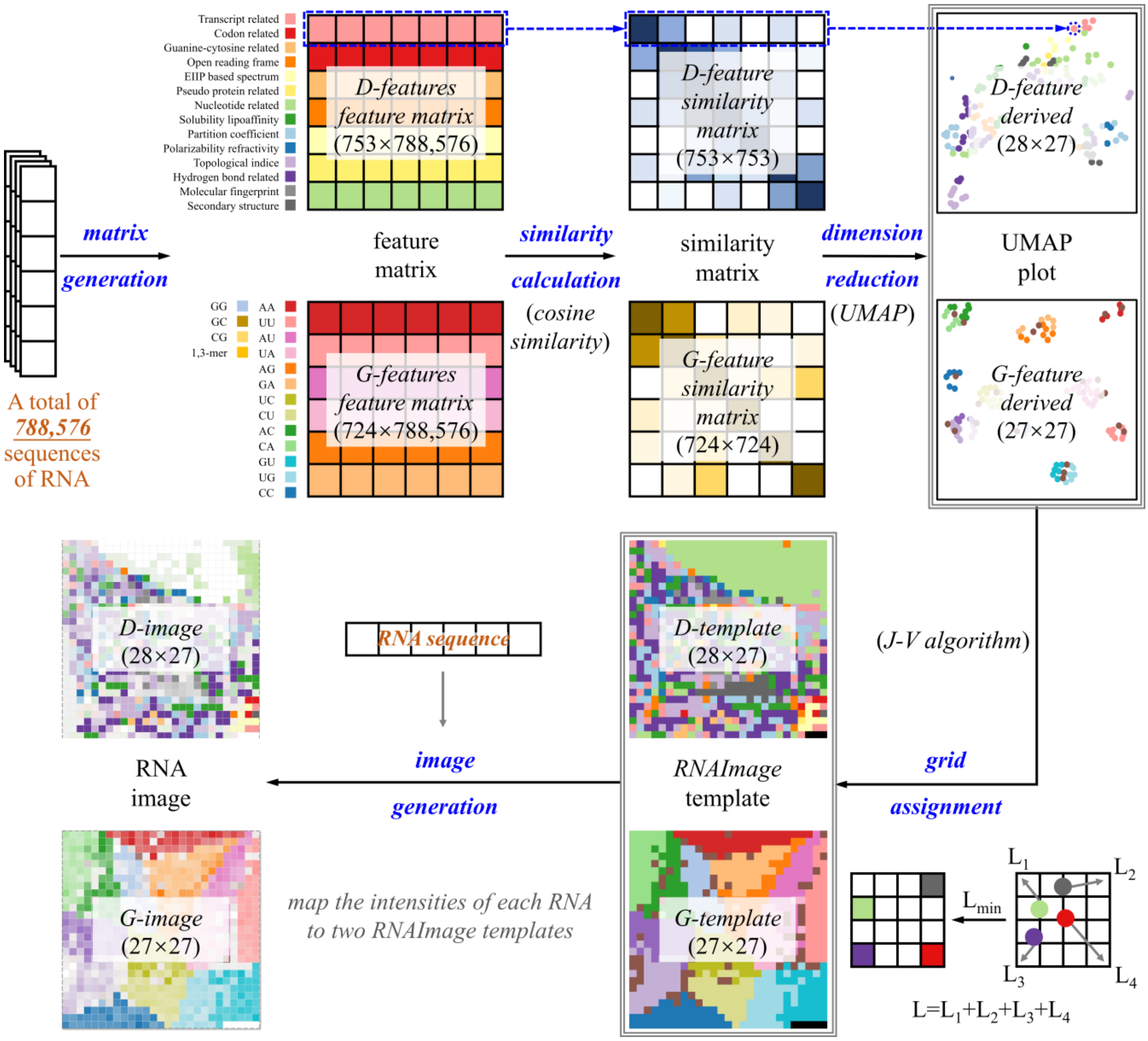

Figure S10. A schematic illustration of the RNA representation using the *RNAImage* strategy proposed in this study. Two template images (*D-template* & *G-template*) were generated using a total of 788,576 RNA sequences collected from established databases (*RefSeq* & *ENCODE*) by sequentially following four procedures, which were from *matrix generation*, to *similarity calculation*, to *dimension reduction*, and finally to *grid assignment*. After the generation of *RNAImage* templates, each RNA sequence was further represented as two images containing abundant feature information.

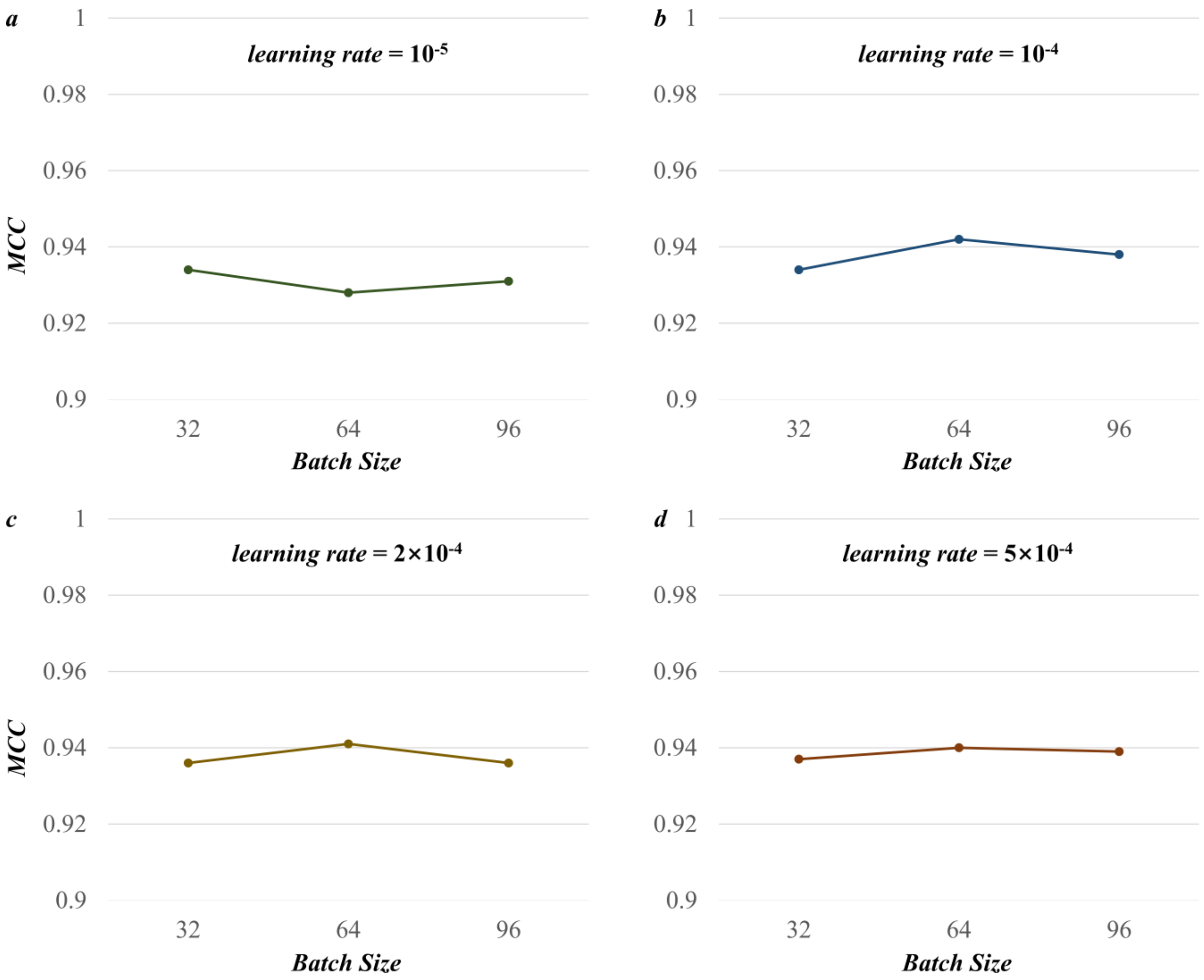

Figure S11. The optimization of hyperparameters used for predicting RNA coding potential. The batch sizes were set as: 32, 64, and 96, and the learning rates were set as: (***a***) 0.00001, (***b***) 0.0001, (***c***) 0.0002, and (***d***) 0.0005.

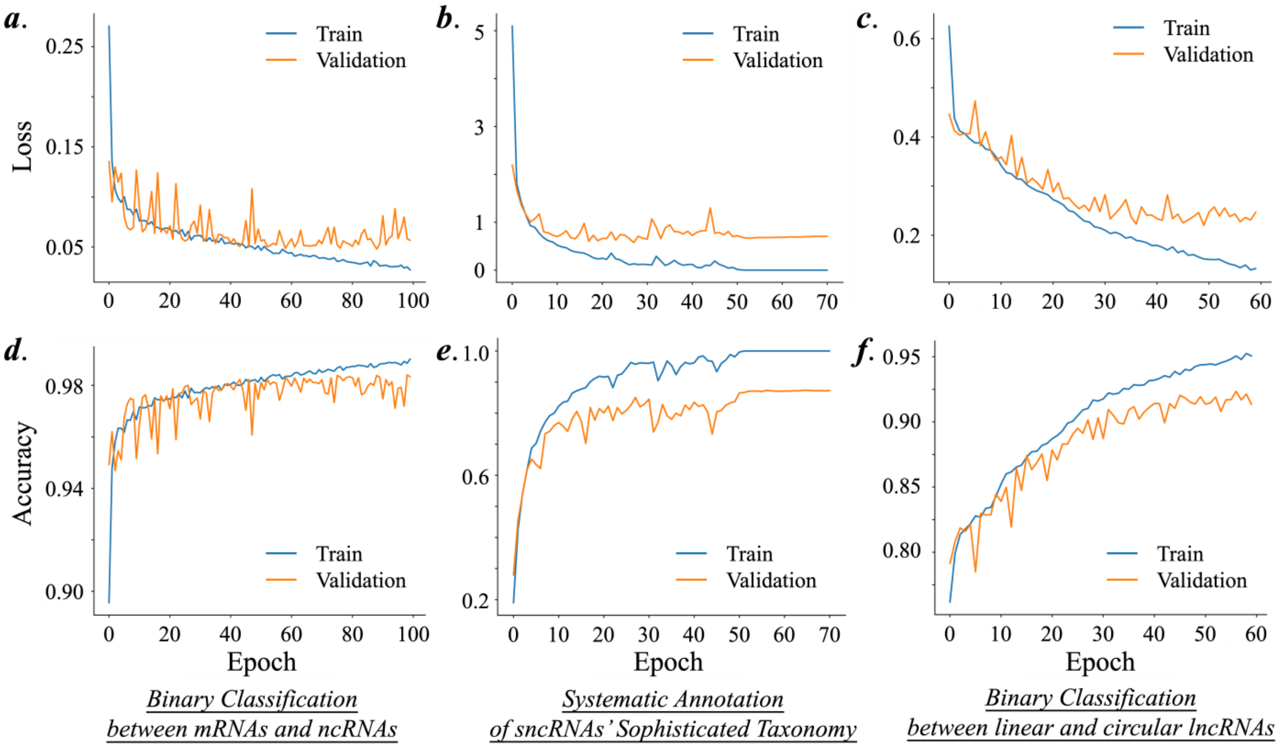

Figure S12. Training and validation accuracy and loss curves across epochs for three classification tasks: (***a***) & (***d***) for RNA coding potential prediction, (***b***) & (***e***) sncRNA taxonomy annotation, and (***c***) & (***f***) circular *vs*. linear lncRNA discrimination.

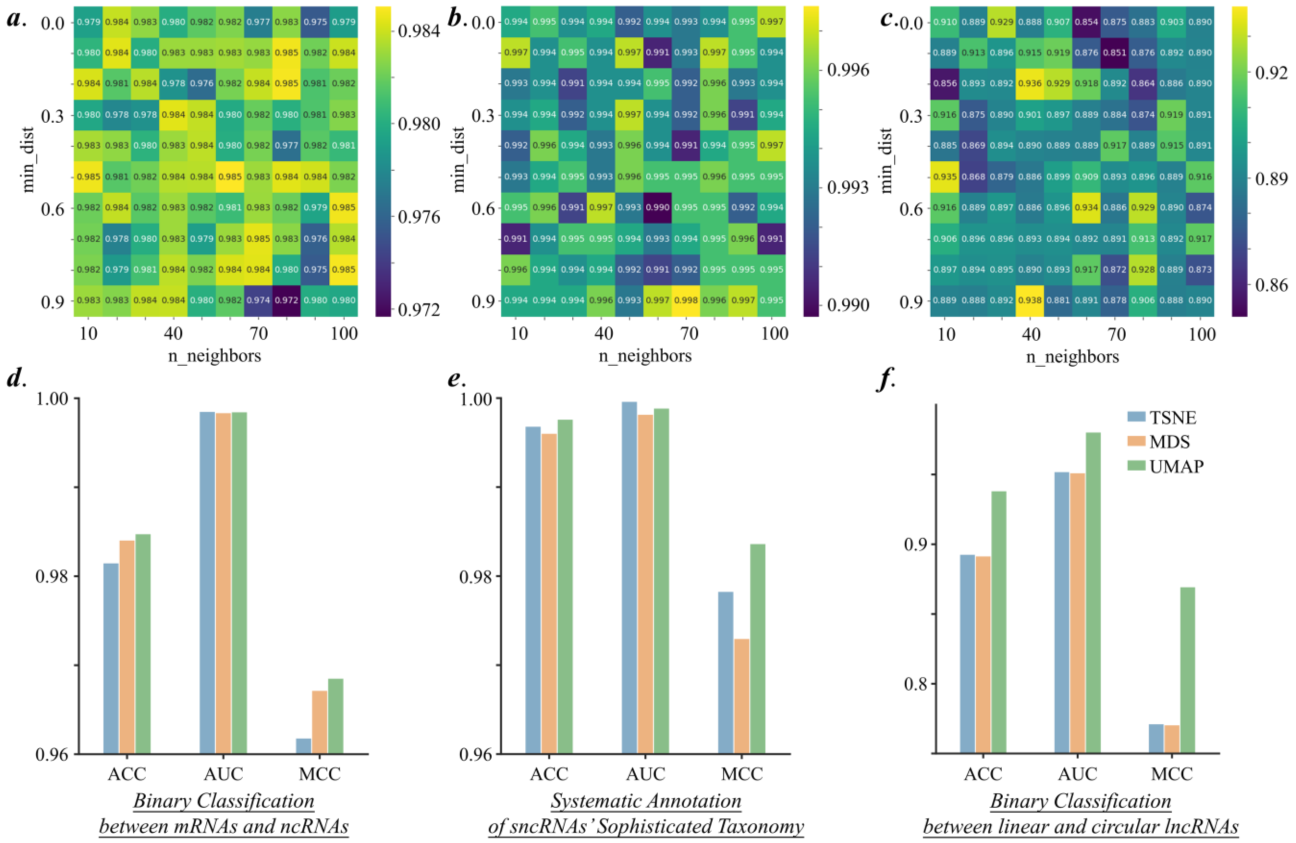

Figure S13. Parameter tuning of UMAP (Uniform Manifold Approximation and Projection) and dimensionality reduction method comparison across three key problems. For each UMAP parameter combination (min_dist: 0.0–0.9, n_neighbors: 10–100), classification accuracy on the validation set was recorded and visualized as heatmaps. In parallel, UMAP was compared against two alternative non-Euclidean dimensionality reduction techniques: t-SNE (t-distributed Stochastic Neighbor Embedding) and MDS (Multidimensional Scaling). (***a***) UMAP parameter tuning & (***d***) dimensionality reduction comparison for RNA coding potential prediction; (***b***) UMAP parameter tuning & (***e***) dimensionality reduction comparison for sncRNA taxonomy annotation; (***c***) UMAP parameter tuning & (***f***) dimensionality reduction comparison for circular *vs*. linear lncRNA discrimination.

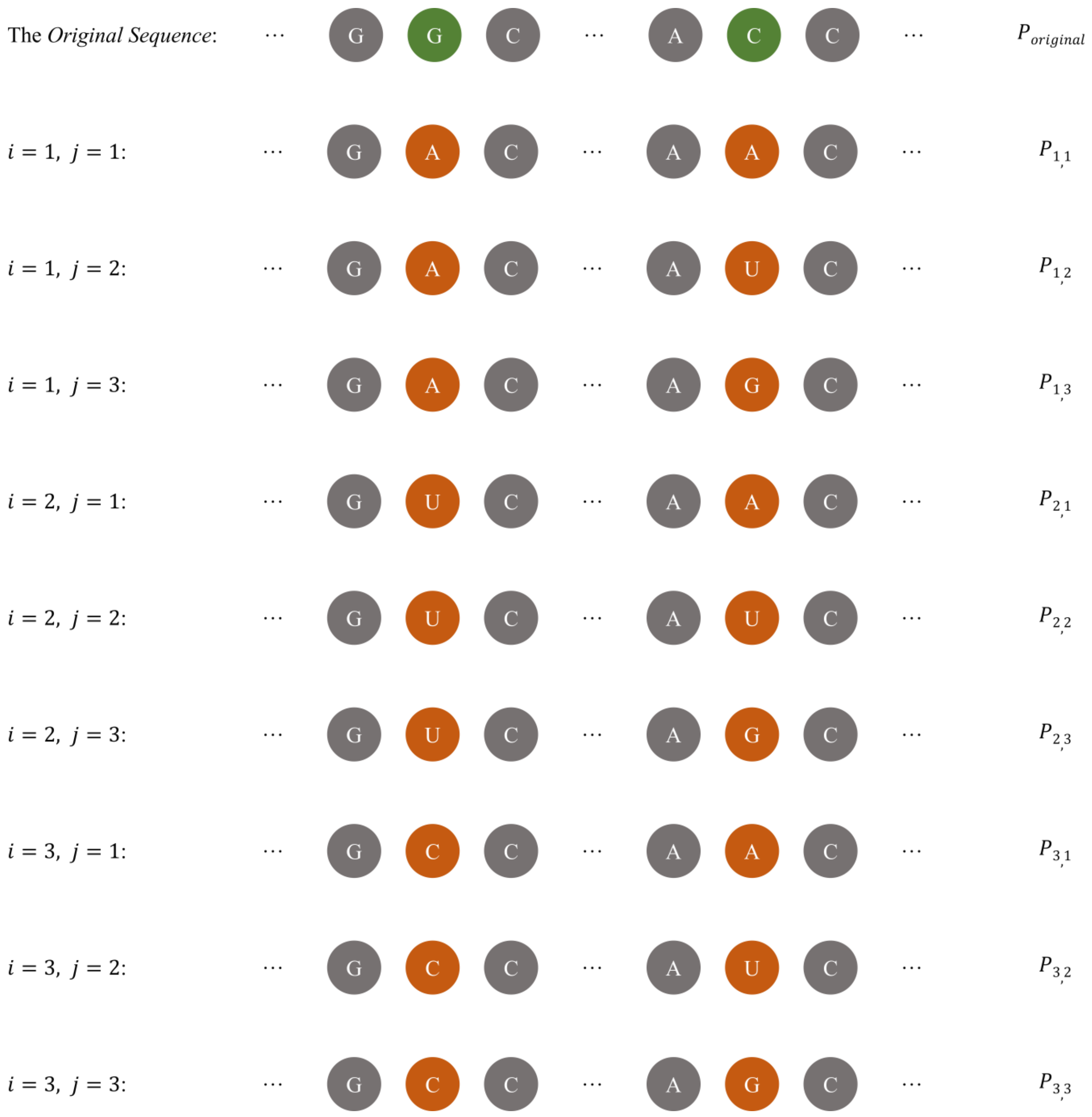

Figure S14. Pairwise mutation analyses of *G-features* for the c-JUN 5' UTR segment. The contribution value of each feature was calculated using the approach described in the *Step 5* of the **Methods** section. The higher the value was, the greater contribution the studied pair made to RNA coding potential.

Method S1. Summary of D-feature Construction

1. Sequence intrinsic descriptors

1.1. Transcript related (all 55 vectors)

1.1.1. Entropy Density Related (20 vectors)

EDP treats RNA sequences as a kind of text, and uses word frequencies commonly used in text research to describe certain properties of RNA. EDP uses the principle of Shannon entropy of artificial languages to deal with short motif frequencies. [1], The short motif used here is a classical 3-mer that converts an RNA sequence into the amino acid sequence of a protein. Therefore, every RNA sequence, no matter how long, will be converted to a frequency result based on the 20 basic amino acids. Then, there are only 20 EDP eigenvalues calculated by a sequence [2, 3]. Here, these 20 features totally calculated on whole RNA transcript based on 20 kinds of nature amino acids were inducted for RNA representation.

1.1.2. Global Descriptor (30 vectors)

Global transcript sequence descriptor encoding feature is to describe the proportion of four different bases, the conversion frequency between each other, and the position in the RNA sequence [4]. The global characteristics of the entire RNA are reflected by the properties and characteristics of the four bases in the RNA sequence. Therefore, the content of the four bases is a 4 feature. There are 6 permutations for the conversion of the four bases. The frequency at which a base occurs at five fixed positions in the entire sequence will form five features [5]. There are 4 bases in total. So, there are a total of 20 encoding features [6]. Here, these 30 features totally were inducted for RNA representation.

1.1.3. RNA length and untranslated region (UTR) related features (5 vectors)

The length of an RNA transcript refers to the total number of nucleotides from the transcription start site to the transcription end site, encompassing both coding and non-coding regions. Within this transcript, the UTR length specifically represents the combined length of the 5′ and 3′ untranslated regions, which do not code for protein but play crucial regulatory roles in mRNA stability, localization, and translation efficiency. The UTR coverage measures the sequencing depth across these untranslated regions, providing insight into how well the UTRs are represented in RNA-seq data. High or uneven UTR coverage can indicate technical biases or biologically relevant variation, such as alternative transcription start or end sites. Together, these metrics help in evaluating transcript structure and expression dynamics in RNA sequencing analyses.

1.2. Open Reading Frame (all 23 vectors)

1.2.1. Basic ORF features (3 vectors)

Basic ORF features are some properties of ORFs in RNA-seq, such as ORF length, ORF coverage, and ORF integrity. These ORF-based features are often used to determine whether RNA transcripts have coding ability [5, 7].

1.2.2. Entropy Density Profiles on ORF (20 vectors)

EDP treats RNA sequences as a kind of text, and uses word frequencies commonly used in text research to describe certain properties of RNA. EDP uses the principle of Shannon entropy of artificial languages to deal with short motif frequencies. [1], The short motif used here is a classical 3-mer that converts an RNA sequence into the amino acid sequence of a protein. Therefore, every RNA sequence, no matter how long, will be converted to a frequency result based on the 20 basic amino acids. Then, there are only 20 EDP eigenvalues calculated by a sequence. In addition to calculating the EDP value for the entire sequence, the EDP can also be calculated only based on the ORF in the sequence [2, 3]. Here, these 20 features totally calculated on ORF based on 20 kinds of nature amino acids were inducted for RNA representation.

1.3. Guanine-cytosine related (7 vectors)

The number of hydrogen bonds between different purines and pyrimidines varies. For example, there are two hydrogen bonds between Adenine and Thymine, and between Adenine and Uracil. Whereas there are three hydrogen bonds between Guanine (G) and Cytosine (C). The more hydrogen bonds in the three-dimensional structure of RNA, the more stable the RNA molecule. The more GC ratio in RNA, the more stable the RNA molecule[8]. The Guanine-cytosine Related includes GC content, first position (GC1), second position (GC2), third position (GC3), the variance of GC contents of first, second and third position of different reading frames. The more detailed definitions can be found in published literature [9]. Some studies have used deep learning methods to identify and annotate lncRNAs based on GC content [10, 11].

1.4 Codon Related Features (all 5 vectors)

1.4.1 Stop Codon Related Features (4 vectors)

As part of the genetic code, a codon generally consists of three consecutive nucleotides. In RNA translation, different codons correspond to specific amino acids. In particular, the start codon and stop codon also control the initiation and termination of RNA translation into protein [12]., which are very important RNA characteristics. Stop codon-related features include the number and frequency of stop codons occurring in the RNA sequence, and the variance in number and frequency between different reading frames [13].

1.4.2. Fickett Score (1 vectors)

The Fickett score is also named the Fickett TESTCODE score. This encoding method, first proposed by Fickett [14], is based on nucleotide composition and codon usage bias composition. The coding potential of the RNA sequence is reflected by calculating the four nucleotide base composition values $A_{composition}$ and position values $A_{position}$. It was obtained by computing four nucleotide base composition values and position values. Composition value was the number of one base in an RNA sequence. The position value is the priority of different types of bases at different positions in the codon. According to the obtained composition value $A_{composition}$ and position value $A_{position}$. The probability value and weight value of different bases can be obtained by looking at the known coding table: $P_{composition(A)}$, $W_{composition(A)}$, $P_{position(A)}$ and $W_{position(A)}$. Then calculate the Fickett score according to the existing formula [15].

2. Physicochemical property-based descriptors

2.1. Pseudo Protein Related (5 vectors)

Non-coding RNA is RNA that cannot be encoded into protein. Pseudoproteins can thus be generated by codon rules. Physicochemical properties of proteins are then calculated based on these pseudoprotein sequences [13]. According to these properties, the potential of this RNA coding into protein can be judged. The properties include the molecular weight (MW) of the pseudopeptide; the theoretical isoelectric point (pI) of the pseudopeptide; the log 10 transformation ratio of pI and Mw ; the overall average of the hydraulicity of the pseudopeptides; the stability of the pseudopeptides [16].

2.2. Nucleotide Related (all 182 vectors)

A total of 136+46 features were calculated based on the physicochemical properties of 38 dinucleotides.

2.2.1. Autocorrelation of Dinucleotide (136 vectors)

The autocorrelation coefficient-encoded features of RNA are encoded by measuring the cross-covariance between different bases of the same attribute. These same properties refer to some physicochemical properties between two nucleotides [17]. Then, for RNA sequences with different lengths, they can be encoded into vectors of the same length according to the same number of physical and chemical properties [17]. The separation distance between two nucleotides is also an important calculation parameter. Based on the existing literatures, there are 38 physical and chemical properties of nucleotides. When the distance is 2, it can be encoded as 38×2 features. Here, N=6 representative physicochemical properties were picked, including PID, BDS, ETI, SKE, WCI, SLI, PID, BDS, ETI, SKE, WCI, SLI, and LAG was preset to 2, thus 60 features were inducted totally [17].

2.2.2. Pseudo Dinucleotide Composition (46 vectors)

Pseudo dinucleotide composition (PseDNC) is not only based on the intrinsic materialization properties of the 38 sequences described above, but also considers continuous local and global information. In this calculation process, there are other parameters. This encoding method takes into account both Parallel correlation PseDNC (PC-PseDNC) and Series correlation PseDNC (SC-PseDNC) [17]. In this calculation process, there is a parameter that represents the highest count level according to the correlation of the series, usually an integer. A value of 2 is commonly used in this study. There is another weighting factor with values ranging from 0 to 1. 18 features were induced totally by PC-PseDNC. In this study, Representative physicochemical properties were picked out, including PID, BDS, ETI, SKE, WCI, SLI, PID, BDS, ETI, SKE, WCI, SLI, and a value of 2 was preset. In such a way, 28 features were inducted totally by SC-PseDNC.

2.3. EIIP-based Spectrum (8 vectors)

Electron-ion interaction pseudopotential (EIIP) values are based on each nucleotide-specific EIIP value (A→0.1260; C→0.1340; G→0.0806; T→0.1335) reflecting the energy of delocalized electrons in the RNA sequence [18]. The EIIP value calculated based on this physicochemical property represents the spectral characteristic of the RNA sequence [18]. After using Fast Fourier Transform, the EIIP value of a nucleotide sequence can be quickly calculated [19, 20]. Here, eight features were inducted from EIIP-based spectrum for RNA representation [13, 18], including the signal value of the third position (peak value), the averaging power of a sequence, the signal/noise ratio, five position values (minimum, the lower quartile, the medium, the upper quartile and the maximum of the sorted power spectrum values).

3. Structure-based features

3.1. Secondary Structure (8 vectors)

This is a multi-scale encoding method proposed by Han et al. based on the formation of secondary structure of RNA sequence, including three properties of secondary structure formation: stability, structural element pairing conditions and structure-nucleotide pairing results. Specifically, stability is the basic structural profile showing the stability of RNA structure, which belongs to the most basic structural properties. The pairing status of structural elements indicates the pairing status of the bases in the sequence. Generally, the bases that can be paired follow the basic Watson-Crick pairing principle, and of course there are many other unusual elements. The structure and pairing relationships of nucleotides in the sequence are the most complex features. These properties represent secondary infrastructure and spatial distance relationships between sequences [21] [18]. Together with MFE, a total of 8 features were inducted including: the percentage of unpaired based on Paired-Unpaired Seq;

the MFE of the nucleotide sequence; the logarithm distance of the nucleotide sequence to lncRNA; the logarithm distance of the nucleotide sequence to pcRNA; the ratio of two distances above; the logarithm distance of the nucleotide sequence to lncRNA; the logarithm distance of the nucleotide sequence to pcRNA; the ratio of two distances above.

3.2 k-mer structure-based descriptors (20 vectors)

For each input RNA sequence, it computes the minimum free energy (MFE) of the predicted secondary structure using RNA.fold(), and normalizes this value by sequence length to obtain the normalized MFE (nMFE). It also calculates the total number of base pairs by counting the opening brackets in the dot-bracket structure. To characterize local structural patterns, the function generates a set of **k-mer structure-based descriptors** by sliding a window of size 1 to 4 along the dot-bracket notation and recording the frequency of all possible structural motifs composed of paired '(' and unpaired '.' symbols. This results in 20 distinct k-mer patterns ($2^2 + 2^4$) whose counts are normalized and concatenated into a fixed-length feature vector. These k-mer structural descriptors capture local folding signatures and secondary structure complexity, and together with the energy-based features, they form a comprehensive feature set suitable for machine learning or statistical modeling of RNA structure-function relationships.

Method S2. The detailed description of 439 encoding descriptors (*D-features*) newly-proposed in this study. Particularly, a total of eleven physicochemical properties & feature groups were considered for the construction of those new *D-features*, which were explicitly described in the following table.

**Feature**	**Detailed Description of Each Encoding Feature**
Lipoaffinity index	a molecular descriptor computed using only 2D data of structure and its estimation is straightforward and computationally inexpensive
Partition coefficient	a concentration ratio of a chemical between two immiscible solvents, typically water (aqueous phase) and octanol (organic phase)
Molar refractivity	a measure of the volume occupied by an atom/group and is dependent on the temperature, the index of refraction, and the pressure
Strong H-donor count	a count of strong hydrogen bond donors having more electronegative the atom attached to hydrogen than weaker H-bond donors
Hydrogen bond basicity	a hydrogen-bond acceptor ability to interact with other hydrogen bond donors and form intermolecular hydrogen bonds
Strong H-acceptor count	a count of strong hydrogen bond acceptors which accept a hydrogen bond through lone pairs of electrons on electronegative atoms
Potential H-bond count	a count of potential hydrogen bond formed in a molecular structure based on the distance between donor and acceptor atoms
Secondary amine count	one of the most common types of molecule fingerprint which is defined as the presence or absence of -NH- in the molecule
Path length oxygens	a topological indices descriptor which is a numerical parameter characterizing its topology and is usually graph invariant
Molecule distance edge	the shortest path between nitrogen atoms in a molecular structure, considering both primary and secondary nitrogen atoms
Polar surface area	the contribution to the molecular surface area of polar atoms (oxygen, nitrogen) and attached hydrogens

The RNA sequence contains four types of nucleotides that are specified by four nucleobases: *Adenine* (A), *Guanine* (G), *Cytosine* (C) and *Uracil* (U). According to those eleven physicochemical properties and feature groups of those four nucleobases (which were explicitly described in the table below), the corresponding values of those properties and groups were calculated based on the *PaDEL-Descriptor* (*J Comput Chem*. 32:1466, 2011). Each nucleobase was divided to two or three group types based on

the values corresponding to each physicochemical property or feature group. As a result, a total of six feature classes were generated, and the parameters adopted in this generation were shown below.

Class Name	Properties	A	C	G	U
Lipoaffinity index	*Lipoaffinity index*	-1.370	-1.767	-2.088	-1.872
	2 group types	1	2	2	2
	3 group types	1	2	3	2
Partition coefficient	*Partition coefficient*	14.395	12.495	14.432	13.600
	2 group types	1	2	1	1
	3 group types	1	2	1	3
Molar refractivity	*Molar refractivity*	44.816	63.122	56.735	62.065
	2 group types	1	2	1	2
Hydrogen bond related	*Strong H-donor count*	5	5	6	5
	2 group types	1	1	2	1
	Hydrogen bond basicity	4.095	4.556	5.224	3.975
	2 group types	1	2	2	1
	Strong H-acceptor count	9	10	11	11
	3 group types	1	2	3	3
	Potential H-bond count	3	4	5	3
	3 group types	1	2	2	3
Molecular fingerprint	*Secondary amine count*	0	0	1	1
	2 group types	1	1	2	2
Topological indices	*Path length oxygens*	18.240	20.764	20.798	23.284
	3 group types	1	2	3	1
	Molecule distance edge	1.040	0.500	1.105	0.000
	2 group types	1	1	1	2
	Polar surface area	195.88	184.95	211.56	175.67
	3 group types	1	1	2	3

For example, lipoaffinity index is a molecular descriptor computed using only 2D information about chemical structure. Lipoaffinity index of these four types of nucleotides was calculated as -1.370, -

1.767, -2.088, -1.872. According to these four values, nucleotides can be divided into two groups, A belongs to one groups and G, C and U belong to the other group. RNA sequence can be represented by 1 and 0 (the bases A is represented as 0, and the bases G, C and U are all represented as 1). In this way, RNA sequence can be represented as a new sequence containing only 0 and 1. Meanwhile, according to the lipoaffinity index of these four nucleotides, they can also be divided into three groups: (1) A, (2) C & U and (3) G. These three groups are replaced by 0, 1, and 2, respectively. In this way, RNA sequence can be represented as a new sequence containing 0, 1, and 2.

RNA sequences derived from physical and structural properties can be encoded by two methods: (1) composition, transition and distribution of RNA sequence, (2) counts of k-mer frequency in RNA sequence. Specifically, when original RNA sequences are divided into two groups based on these physical and structural properties, the composition of each character in the new generated sequence are 0 and 1 and the encoding features are two elements. Transition describes the percent frequency with conversion of two characters between adjacent positions. Two kinds of characters would have one mutation: 01 or 10. The characters distribution describes the content of two characters at the five relative positions along the new RNA sequence of each character, including the 0%, 25%, 50%, 75% and 100%. According to this encoding method, 13 vectors can be obtained.

Using the second encoding method, the 1-mer, 2-mer, and 3-mer frequency of RNA are calculated to form the sequence encoding vectors with $2^1 + 2^2 + 2^3 = 14$ vectors. Twenty-seven vectors can be obtained by two methods for encoding new generated sequences divided into two groups according to physicochemical and structural properties. On another scale, when original RNA sequences are divided into three groups based on these physical and structural properties, 21 (3+3+15) vectors can be obtained by calculating composition, transition and distribution for sequence. 39 ($3^1+3^2+3^3$) vectors can be obtained by counting k-mer frequency. In the process of encoding feature according to the above two encoding methods, there are two types of different encoding duplications. On the one hand, encoding duplication exists when nucleotides divided into two groups and three groups according to the same property. For example, A is one separate group according to lipoaffinity index whatever nucleotides are divided into two groups or three groups. On the other hand, encoding duplication exists when nucleotides are divided into the same group according to the different property. For instance, A is one separate group whether based on count for potential H-bonds of path length or lipoaffinity index when nucleotides are divided into three groups. When a single base is classified as one separate group according to different properties or different classes, there is duplication in the encoding result because there is a step to calculate the content in both methods. Therefore, it is necessary to remove the duplication based on all eleven physical and chemical properties and to obtain non-repeated encoding feature for each property, as shown in **Figure 2**.

Reference

[1] C.E. Shannon, The mathematical theory of communication. 1963, MD Comput, 14 (1997) 306-317.

[2] Z. Ouyang, H. Zhu, J. Wang, Z.S. She, Multivariate entropy distance method for prokaryotic gene identification, J Bioinform Comput Biol, 2 (2004) 353-373.

[3] Y. Liu, J. Guo, G. Hu, H. Zhu, Gene prediction in metagenomic fragments based on the SVM algorithm, BMC Bioinformatics, 14 Suppl 5 (2013) S12.

[4] L.Y. Han, C.Z. Cai, Z.L. Ji, Z.W. Cao, J. Cui, Y.Z. Chen, Predicting functional family of novel enzymes irrespective of sequence similarity: a statistical learning approach, Nucleic Acids Res, 32 (2004) 6437-6444.

[5] X. Tong, S. Liu, CPPred: coding potential prediction based on the global description of RNA sequence, Nucleic Acids Res, 47 (2019) e43.

[6] G.-S. Han, Z. Yu, V. Anh, R. Chan, Distinguishing Coding from Non-coding Sequences in a Prokaryote Complete Genome Based on the Global Descriptor, 2009.

[7] Y.J. Kang, D.C. Yang, L. Kong, M. Hou, Y.Q. Meng, L. Wei, G. Gao, CPC2: a fast and accurate coding potential calculator based on sequence intrinsic features, Nucleic Acids Res, 45 (2017) W12-W16.

[8] P. Yakovchuk, E. Protozanova, M.D. Frank-Kamenetskii, Base-stacking and base-pairing contributions into thermal stability of the DNA double helix, Nucleic Acids Res, 34 (2006) 564-574.

[9] S. Liu, X. Zhao, G. Zhang, W. Li, F. Liu, S. Liu, W. Zhang, PredLnc-GFStack: A Global Sequence Feature Based on a Stacked Ensemble Learning Method for Predicting lncRNAs from Transcripts, Genes (Basel), 10 (2019).

[10] J.M. Kirk, S.O. Kim, K. Inoue, M.J. Smola, D.M. Lee, M.D. Schertzer, J.S. Wooten, A.R. Baker, D. Sprague, D.W. Collins, C.R. Horning, S. Wang, Q. Chen, K.M. Weeks, P.J. Mucha, J.M. Calabrese, Functional classification of long non-coding RNAs by k-mer content, Nat Genet, 50 (2018) 1474-1482.

[11] C. Yang, L. Yang, M. Zhou, H. Xie, C. Zhang, M.D. Wang, H. Zhu, LncADeep: an ab initio lncRNA identification and functional annotation tool based on deep learning, Bioinformatics, 34 (2018) 3825-3834.

[12] C. Touriol, S. Bornes, S. Bonnal, S. Audigier, H. Prats, A.C. Prats, S. Vagner, Generation of protein isoform diversity by alternative initiation of translation at non-AUG codons, Biol Cell, 95 (2003) 169-178.

[13] S. Yang, Y. Wang, S. Zhang, X. Hu, Q. Ma, Y. Tian, NCResNet: Noncoding Ribonucleic Acid Prediction Based on a Deep Resident Network of Ribonucleic Acid Sequences, Front Genet, 11 (2020) 90.

[14] J.W. Fickett, Recognition of protein coding regions in DNA sequences, Nucleic Acids Res, 10 (1982) 5303-5318.

[15] L. Wang, H.J. Park, S. Dasari, S. Wang, J.P. Kocher, W. Li, CPAT: coding-potential assessment tool using an alignment-free logistic regression model, Nucleic Acids Res, 41 (2013) e74.

[16] P.J. Cock, T. Antao, J.T. Chang, B.A. Chapman, C.J. Cox, A. Dalke, I. Friedberg, T. Hamelryck, F. Kauff, B. Wilczynski, M.J. de Hoon, Biopython: freely available Python tools for computational molecular biology and bioinformatics, Bioinformatics, 25 (2009) 1422-1423.

[17] B. Liu, F. Liu, L. Fang, X. Wang, K.C. Chou, repDNA: a python package to generate various modes of feature vectors for DNA sequences by incorporating user-defined physicochemical properties and sequence-order effects, Bioinformatics, 31 (2015) 1307-1309.

[18] S. Han, Y. Liang, Q. Ma, Y. Xu, Y. Zhang, W. Du, C. Wang, Y. Li, LncFinder: an integrated platform for long non-coding RNA identification utilizing sequence intrinsic composition, structural information and physicochemical property, Brief Bioinform, 20 (2019) 2009-2027.

[19] A.A. Tsonis, J.B. Elsner, P.A. Tsonis, Periodicity in DNA coding sequences: implications in gene evolution, J Theor Biol, 151 (1991) 323-331.

[20] S. Tiwari, S. Ramachandran, A. Bhattacharya, S. Bhattacharya, R. Ramaswamy, Prediction of probable genes by Fourier analysis of genomic sequences, Comput Appl Biosci, 13 (1997) 263-270.

[21] D. Charif, J. Lobry, SeqinR 1.0-2: A Contributed Package to the R Project for Statistical Computing Devoted to Biological Sequences Retrieval and Analysis, 2007, pp. 207-232.
